# Seven interferon gamma response genes serve as a prognostic risk signature that correlates with immune infiltration in lung adenocarcinoma

**DOI:** 10.18632/aging.202831

**Published:** 2021-04-04

**Authors:** Boyang Yao, Lixin Wang, Heyong Wang, Jinxia Bao, Qiwen Li, Fengzhi Yu, Wenjing Zhu, Li Zhang, Wang Li, Zhan Gu, Ke Fei, Peng Zhang, Fan Zhang, Xiaoying Huang

**Affiliations:** 1 Division of Pulmonary Medicine, The First Affiliated Hospital of Wenzhou Medical University, Key Laboratory of Heart and Lung, Wenzhou 325000, Zhejiang, China; 2 Department of Thoracic Surgery, Shanghai Pulmonary Hospital, Tongji University School of Life Science and Technology, Shanghai 200433, China; 3 Clinical Translational Research Center, Shanghai Pulmonary Hospital, Tongji University School of Medicine, Shanghai 200433, China; 4 Department of Traditional Chinese Medicine, Shanghai Pulmonary Hospital, Tongji University School of Medicine, Shanghai 200433, China; 5 College of Biological and Environmental Engineering, Binzhou University, Binzhou 256600, Shandong, China

**Keywords:** interferon gamma response gene, lung adenocarcinoma, risk signature, prognostic markers, immune infiltration

## Abstract

Interferon-gamma (IFN-γ) plays a complex role in modulating tumor microenvironment during lung adenocarcinoma (LUAD) development. In order to define the role of IFN-γ response genes in LUAD progression, we characterized the gene expression, mutation profile, protein-protein interaction of 24 IFN-γ response genes, which exhibited significant hazard ratio in overall survival. Two subgroups of LUAD from the TCGA cohort, which showed significant difference in the survival rate, were identified based on the expression of these genes. Furthermore, LASSO penalized cox regression model was used to derive a risk signature comprising seven IFN-γ response genes, including CD74, CSF2RB, PTPN6, MT2A, NMI, LATS2, and PFKP, which can serve as an independent prognostic predictor of LUAD. The risk signature was validated in an independent LUAD cohort. The high risk group is enriched with genes regulating cell cycle and DNA replication, as well as a high level of pro-tumor immune cells. In addition, the risk score is negatively correlated with the expression of immune metagenes, but positively correlated with DNA damage repair genes. Our findings reveal that seven-gene risk signature can be a valuable prognostic predictor for LUAD, and they are crucial participants in tumor microenvironment of LUAD.

## INTRODUCTION

IFN-γ is a key cytokine produced by CD4 T helper cells, CD8 cytotoxic T cells [1, 2], natural killer (NK), natural killer T cells (NKT) cells [3], and, to a less extent, by B cells [4], and professional antigen-presenting cells (APCs) in the tumor microenvironment. It plays an important role in coordinating tumor related immune response (10). Its expression is induced by cytokines, including IL-2, IL-12 [5], IL-15 [6], IL-18 [7], and type I interferon [8].

Under physiological conditions, endogenous IFN-γ is vital to many biological processes, including regulation of immune cell functions [9, 10], maintenance of the hematopoietic stem cell niche [10], formation of bone [11], anti-viral host defense. IFN-γ upregulates the expression of MHC class I and genes required for antigen processing to enhance tumor immunogenicity [12]. Therefore, IFN-γ enhances tumor recognition by tumor-specific cytotoxic T lymphocytes, which promotes tumor rejection. Loss of IFN-γ sensitivity is associated with an increased tumor incidence in animals treated with the chemical carcinogen. In humans, functional loss of the tumor suppressor IRF-1, a critical mediator of IFN-γ signaling, is associated with leukemia or gastric cancer [13, 14].

IFN-γ can also have inhibitory immune-regulatory effects on autoimmune [15], or antitumor responses [16]. IFN-γ from lymphocytes enhances the expression of PD-L1 and PD-L2 in tumor and stromal cells, which bind PD-1 on tumor-infiltrating T cells to suppress the cytotoxic response and promotes cancer progression [17, 18]. IFN-γ can also drive the up-regulation of other negative regulatory immune checkpoint molecules such as IDO1 within the tumor microenvironment [19]. Tumor is adapted to take advantage of this positive or negative immune signaling feedback loop to develop and progress. In addition, interferon-stimulated genes are involved in cross-resistance to radiotherapy in tamoxifen-resistant breast cancer [20].

IFN-γ pleiotropic functions are mediated by cell-specific expression of about 200 IFN-γ-regulated genes that include inflammatory signaling molecules, programmed cell death or cell cycle regulators, proteins involved in antigen presentation, and transcriptional factors, such as, major histocompatibility complex (MHC) class I and class II molecules, IRFs, Fc-gamma receptor (FCGR), GBPs (guanylate-binding proteins) and antiviral proteins like PKR, OAS proteins [21, 22]. Characterizing cellular targets of IFN-γ is critical for its prognostic or therapeutic application, particularly in cancers where this cytokine can induce both anti- or pro-tumorigenic effects.

Lung cancer is the leading cause of cancer-related death around the world. Lung adenocarcinoma (LUAD), one of the most frequently observed lung cancer subtypes, has distinct cellular and mutational landscapes with complex immune contexture [23]. Emerging evidence supports that tumor microenvironment impacts LUAD progression and clinical outcome [24]. Nonetheless, no existing study has comprehensively analyzed and screened interferon gamma response genes as the risk signature for LUAD prognosis, or its correlation with various clinical and pathological features. In this study, we systematically profiled the expression and mutation profile, protein-protein interaction of key interferon gamma response genes in LUAD using RNA-seq data from The Cancer Genome Atlas (TCGA) database. Using consensus clustering based on interferon gamma response genes expression profile, we identified and characterized 2 clinically and molecularly distinct LUAD subtypes; furthermore, using Lasso penalized cox regression analysis, a risk signature comprising seven interferon gamma response genes was constructed. The accuracy and sensitivity of the risk signature in prognosis was successfully validated by 2 independent LUAD cohorts from the GEO database. In addition, the high risk group contains immune repressed features, and is enriched with biological pathways including cell cycle, DNA replication, and DNA damage repair. It also has different immune checkpoint gene expression profiles, compared to the low risk group. Overall, our findings could be valuable in predicting clinical outcome and guiding immunotherapy of lung cancer.

## RESULTS

### Transcriptional and genetic alterations of 24 interferon gamma response genes in LUAD

The flow chart of this study was presented (Supplementary Figure 1). We chose the gene set, ‘HALLMARK_INTERFERON_GAMMA_RESPONSE’, for our analysis, which include 200 genes up-regulated in response to interferon gamma, from the molecular signature database of GSEA (https://www.gsea-msigdb.org/). We used TCGA LUAD data as our training set for the risk signature construction. The clinicopathological information of TCGA LUAD patients is summarized (Supplementary Table 1A). Based on initial univariate cox analysis, 24 interferon gamma response genes, which had statistically significant hazard ratio (HR) related to patient overall survival, were used in the subsequent study.

**Figure 1:**
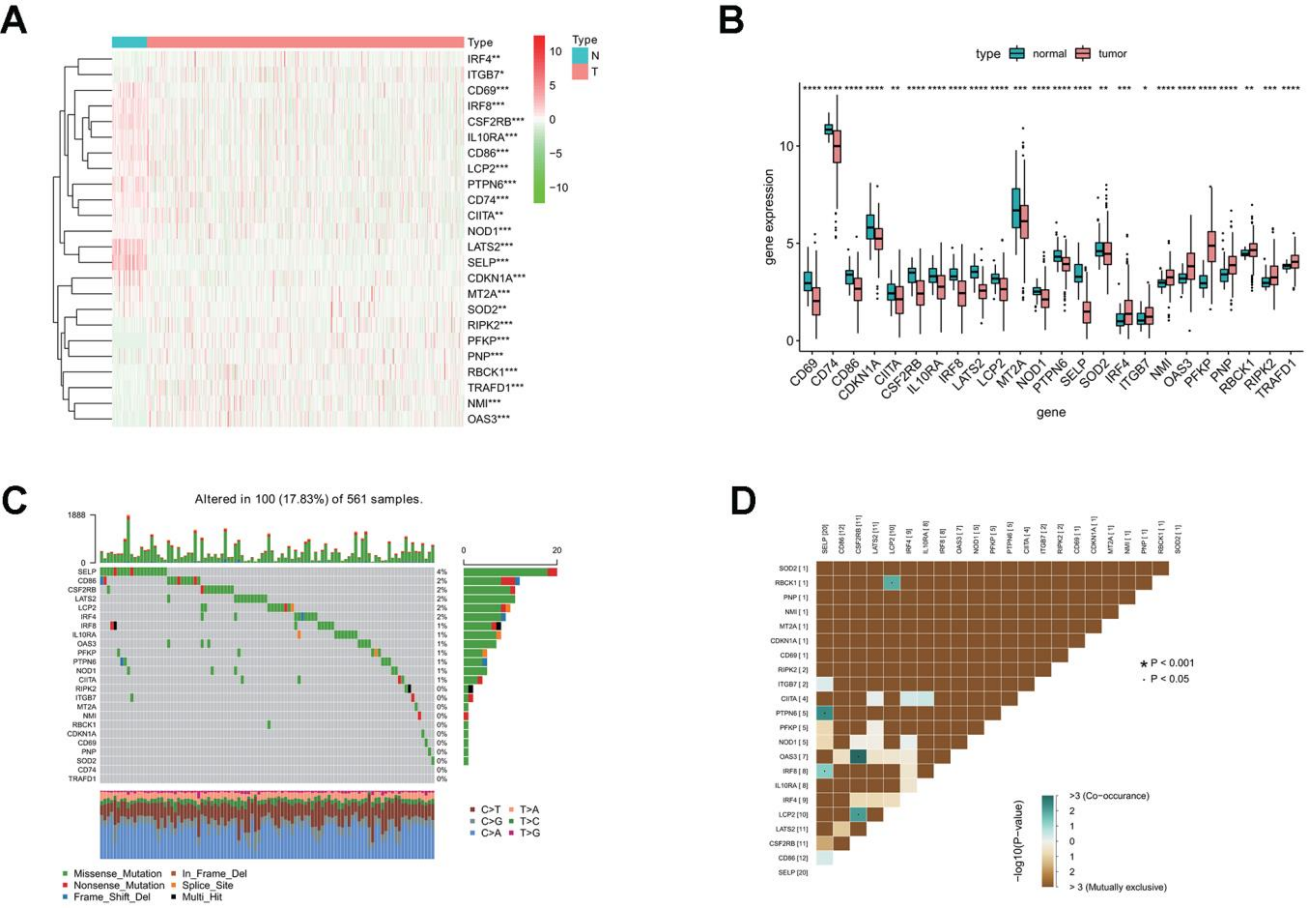
**Differential expression of interferon gamma response genes between tumor and normal tissues in the TCGA LUAD cohort.** (**A**, **B**) Differential expression of interferon gamma response genes between tumor and normal tissues in the TCGA LUAD cohort is presented in the heatmap (**A**) and the box plot (**B**). ***P<0.001 (normal vs. tumor tissues). N, normal; T, tumor. (**C**) The mutation landscape of 24 interferon gamma response genes in 561 patients from the TCGA LUAD cohort. The middle panel depicts the gene mutation patterns across each sample with different mutation type colored differently. The mutation frequency in each gene is listed on the right of the middle panel, total mutation burden for each sample is shown in the upper barplot, the proportion of each mutation type of genes is shown on the right barplot. The fraction of nucleotide conversions in each sample is indicated by the stacked barplot below. (**D**) The correlation coefficient analysis of mutation co-occurrence in 24 interferon gamma response genes from the TCGA LUAD cohort.

To characterize these genes, we compared the expression profile of 24 interferon gamma response genes between tumor and normal samples, and presented the results in the heatmap (Figure 1A) and box plots (Figure 1B), which showed that, the expression of 15 interferon gamma response genes was markedly downregulated in tumor samples, including CD69, CD74, CD86, CDKN1A, CIITA, CSF2RB, IL10RA, IRF8, LATS2, LCP2, MT2A, NOD1, PTPN6, SELP, SOD2, while the expression of 9 genes was markedly upregulated in LUAD, including IRF4, ITGB7, NMI, OAS3, PFKP, PNP, RBCK1, RIPK2, and TRAFD1. The differential gene expression analysis results were summarized (Supplementary Table 2). Among TCGA LUAD samples, 101 had mutations in these genes, with frequency of 18%. It was found that SELP exhibited the highest mutation frequency followed by CD86, CSF2RB, LATS2, LCP2, IRF4, and IRF8, etc, while CD74 and TRAFD1did not show any mutations in these samples (Figure 1C). Further analyses revealed a significant mutation co-occurrence relationship between SELP and PTPN6, SELP and IRF8, OAS3 and CSF2RB1, LCP2 and CSF2RB1, along with LCP2 and RBCK1 (Figure 1D).

Overall, the above analyses revealed a highly heterogeneous landscape of genetic and transcriptional alteration in interferon gamma response genes between tumor and normal samples, implying that these genes may play a crucial role in the tumor occurrence and progression.

### Gene expression correlation and protein-protein interaction among 24 interferon gamma response genes

**Figure 2:**
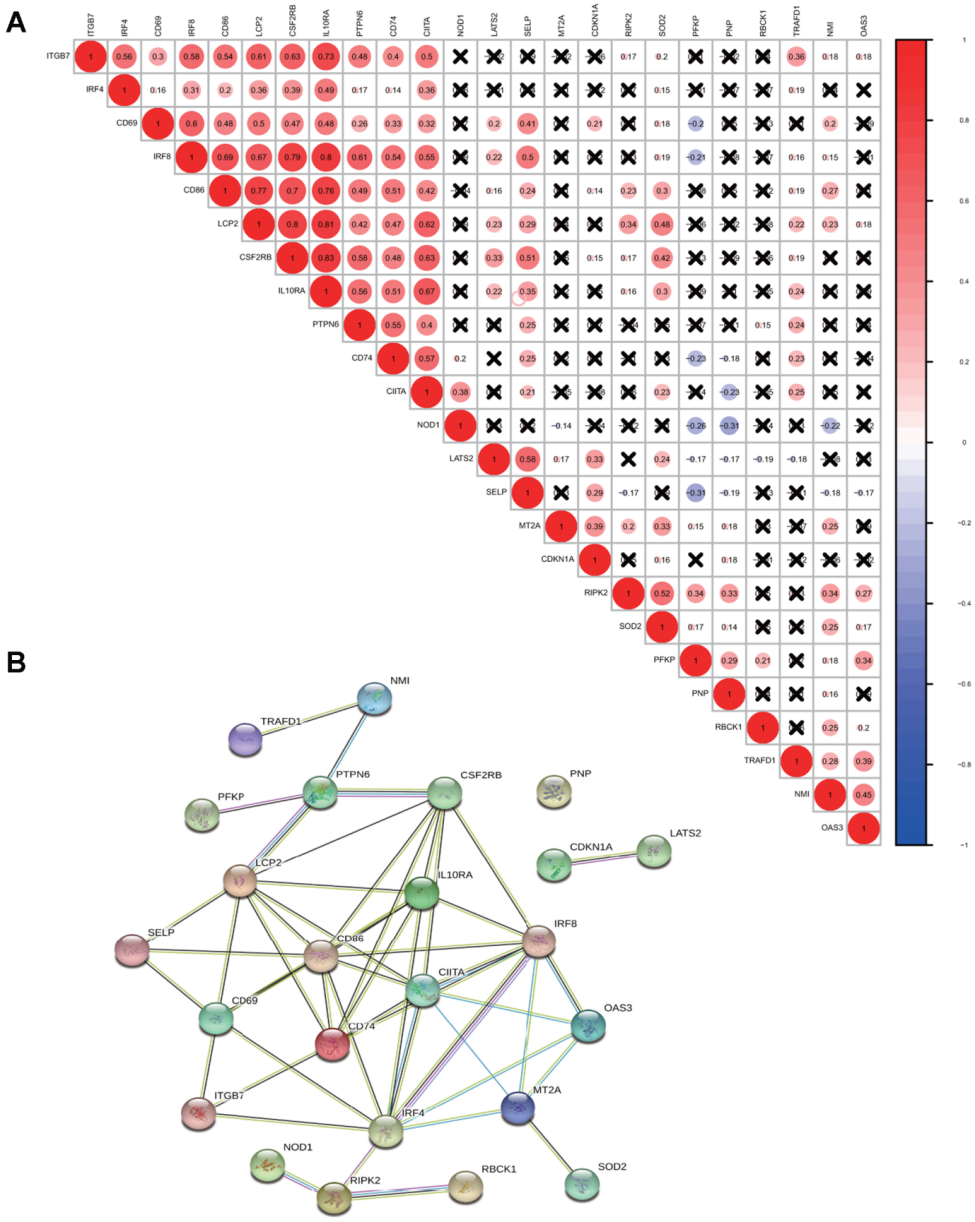
**Correlation and interaction among interferon gamma response genes.** (**A**) Spearman correlation analysis of the expression among 24 interferon gamma response genes from the TCGA LUAD cohort. (**B**) Protein-protein interactions among 24 interferon gamma response genes.

For a better understanding of interactions among these 24 interferon gamma response genes, the correlation in gene expression was calculated (Figure 2A). Clearly, ITGB7 and IRF4 were positively correlated with each other, CD69, IRF8, CD86, LCP2, CSF2RB, IL10RA, PTPN6, CD74, CIITA were positively correlated with each other. SELP exhibited a significantly positive correlation with LATS2. TRAFD1, NMI, and OAS3 positively correlated with each other. In addition, NOD1 were negatively correlated with PFKP, PNP, and NMI, while PFKP were negatively correlated with CD74, NOD1, and SELP. Of note, the correlation between IL10RA and IRF8 (0.8), CD86 (0.7), LCP2 (0.81) and CSF2RB (0.83), ranked top among all correlations.

Protein-protein interaction among them was also analyzed (Figure 2B). The interactions between these proteins were supported by experimental assays (pink lines), the curated databases (blue lines), co-expression (black lines), or text mining (olive green lines). In addition, LCP2, CSF2RB, CD69, CD86, CD74, IL10RA, IRF4, IRF8, CIITA, MT2A are connected with at least four other proteins, suggesting that these proteins may regulate each other. However, CDKN1A and LATS2 connects only with each other, while PNP appears to have no connection. This may suggest that they are regulated by proteins other than those under study. Overall, the protein-protein interaction analysis among 24 interferon gamma response genes indicates that interferon gamma response pathway is tightly regulated process.

### Consensus clustering analysis based on expression of interferon gamma response genes

**Figure 3:**
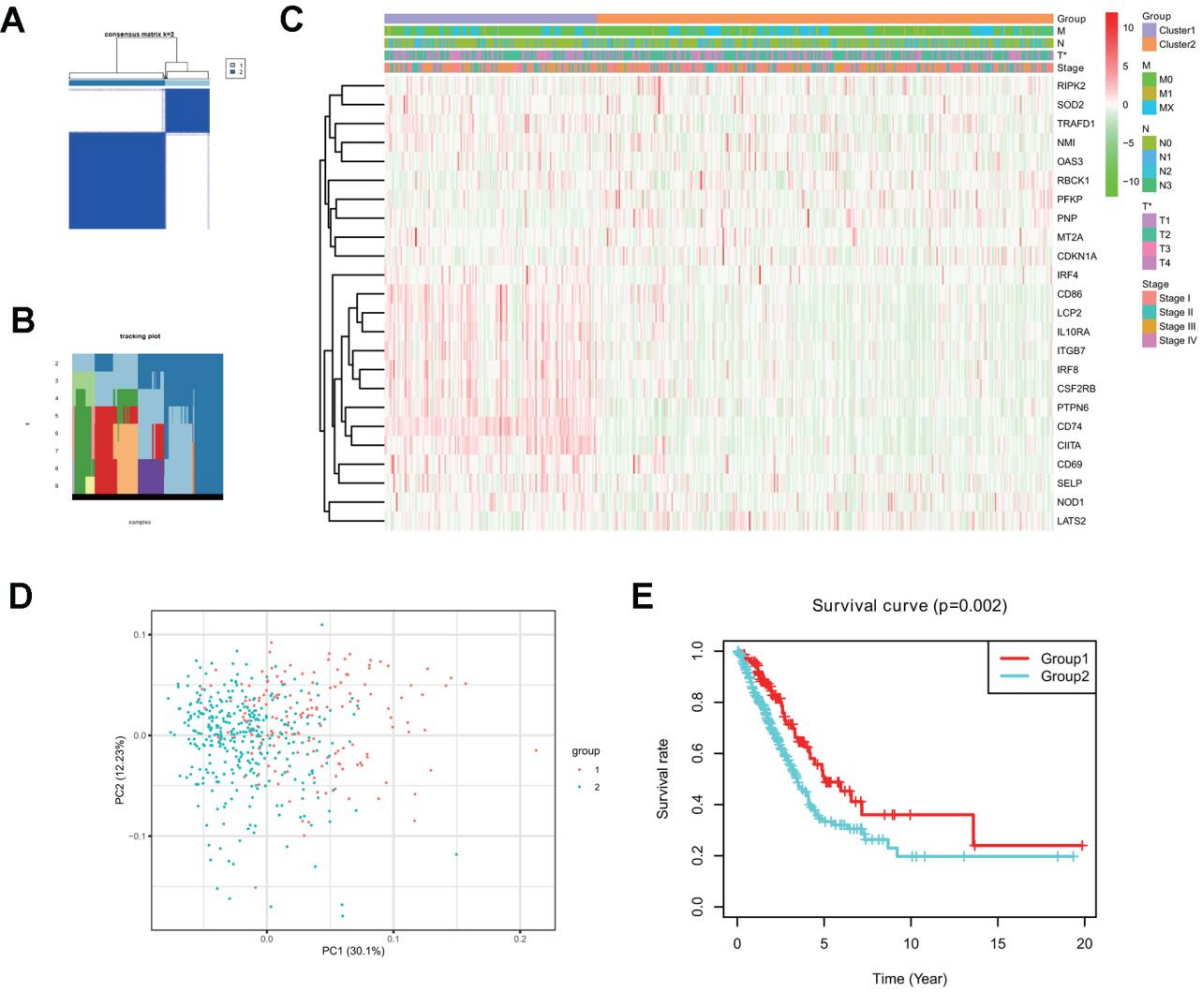
**Two clusters in the TCGA LUAD cohort with distinct clinical outcomes and clinicopathological features identified by consensus clustering.** (**A**) Consensus clustering matrix at k=2. (**B**) Tracking plot at k=2 to k=10 by consensus clustering. (**C**) Heatmap of interferon gamma response gene expression for two clusters (1 and 2) based on consensus clustering of TCGA LUAD tumor samples, together with the clinical and pathological features (T, N, M, or stage). (**D**) Principal component analysis (PC1 vs. PC2) of 24 interferon gamma response gene expression pattern in the TCGA LUAD cohort. Two clusters are marked with colors (green and red). (**E**) Kaplan-Meier overall survival rate curves for two clusters in the TCGA LUAD cohorts. P=0.002 (group 1 vs. group 2).

Consensus clustering analysis indicates that LUAD patients in the TCGA cohort can be classified into two clusters (Figure 3A, 3B), with the range of empirical cumulative distribution (CDF) presented for k = 2 to k = 9 (Supplementary Figure 2A–2J). Therefore, TCGA LUAD cohort was grouped into two clusters based on two fully distinct expression patterns of 24 interferon gamma response genes.

The clinical features between two clusters (clusters 1 and 2) were presented in the heatmap (Figure 3C). Cluster 2 was characterized by the decreased expression of IRF4, CD86, LCP2, IL10RA, ITGB7, IRF8, CSF2RB, PTPN6, CD74, CIITA, CD69, SELP, and NOD1, and the increased expression of other genes, including RIPK2, SOD2, TRAFD1, NM1, OAS3, RBCK1, PFKP, PNP, MT2A, and CDKN1A. Furthermore, cluster 2 was significantly associated with late T stage (P < 0.05).

Next, principle component analysis (PCA) was carried for comparing gene expression patterns between two clusters. Our PCA analysis revealed that these two clusters were markedly different (Figure 3D). finally, cluster 2 had a markedly reduced overall survival rate, as compared with that in cluster1 (P = 0.002) (Figure 3E).

The above analysis indicates that distinct expression patterns of interferon gamma response genes may serve as prognostic markers in LUAD patient survival.

### Identification of a risk signature comprising of 7 interferon gamma response genes in LUAD

**Figure 4:**
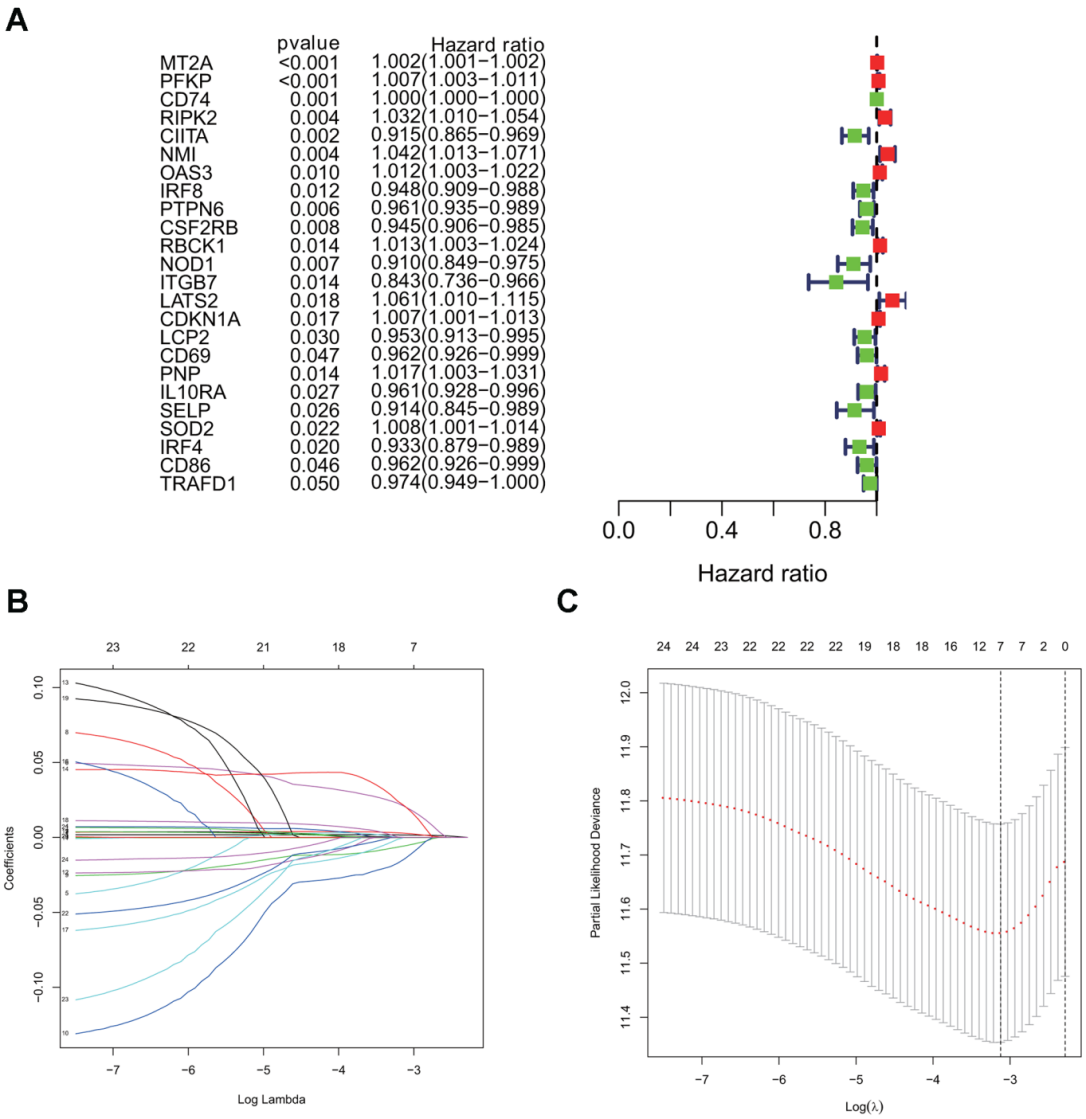
**Univariate cox and LASSO Cox regression analysis for overall survival related interferon gamma response genes.** (**A**) Forest plots showing the prognostic value detection of various clinical features and the risk score, in which the HRs, corresponding 95% confidence intervals, and p values are displayed. (**B**) LASSO regression analysis was used to calculate the coefficient of interferon gamma response genes. (**C**) Seven genes were selected as active covariates to determine the prognostic value after 10-fold cross-validation for the LASSO model.

Using the univariate cox regression model, we analyzed the prognostic value of 24 interferon gamma response genes in the TCGA cohort (P<0.05; Figure 4A). Among them, 14 genes, including CD74, CIITA, IRF8, PTPN6, CSF2RB, NOD1, ITGB7, LCP2, CD69, IL10RA, SELP, IRF4, CD86, and TRAFD1, were the protective genes with a hazard ratio (HR) of less than 1, while 10 genes, including MT2A, PFKP, RIPK2, NMI, OAS3, RBCK1, LATS2, CDKN1A, PNP, and SOD2, were the risk genes with a HR of more than 1.

**Figure 5:**
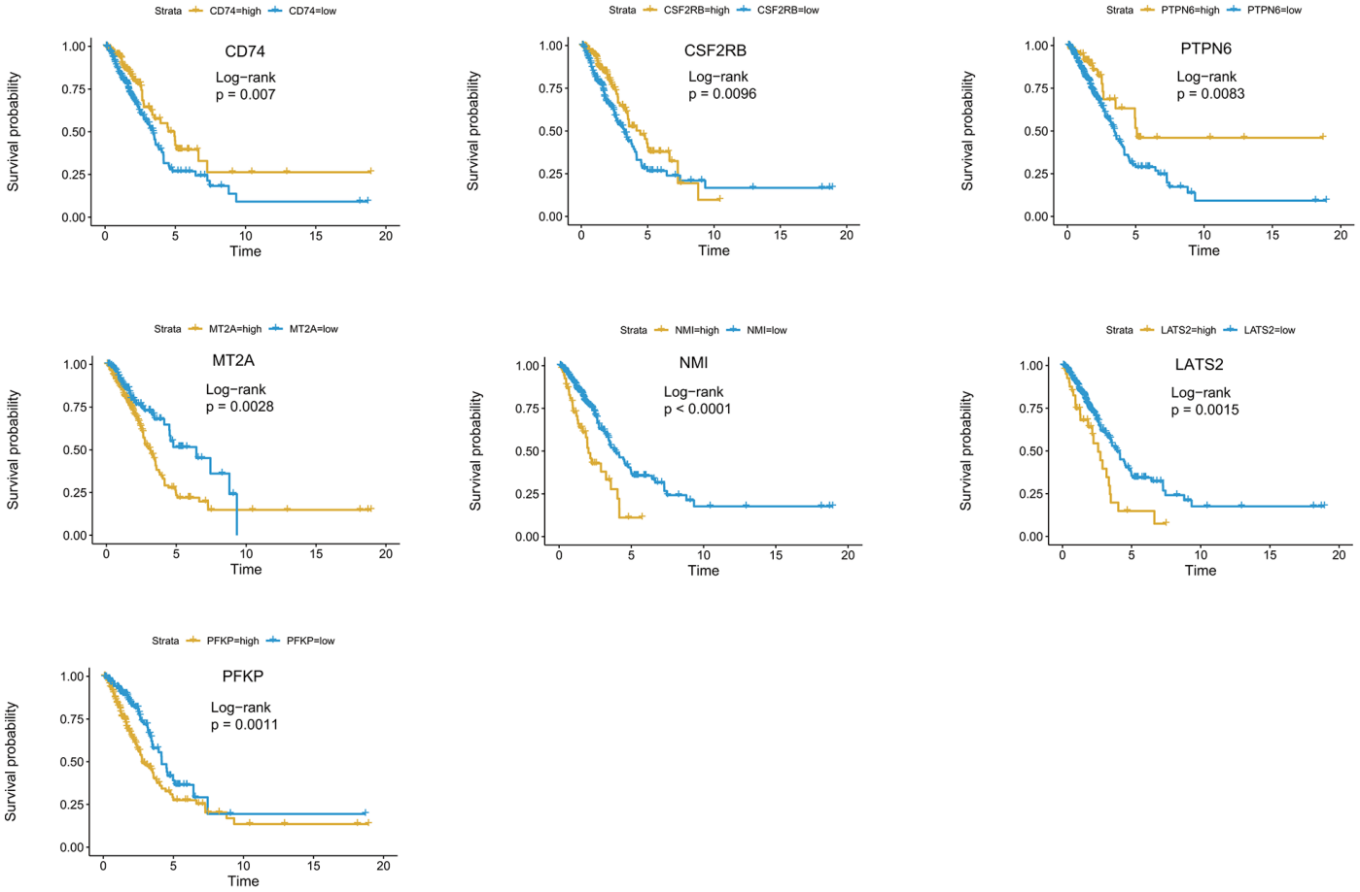
**Kaplan-Meier survival curve analysis of seven genes in the risk signature.** The survival curves and risk tables of seven genes in the TCGA LUAD cohort were presented with the log-rank test results. Among them, high expression of CD74, CSF2RB, or PTPN6 is associated with favorable prognosis, while high expression of MT2A, NMI, LATS2, or PFKP, is associated with poor prognosis in LUAD.

For a more precise prediction of LUAD prognosis using interferon gamma response genes, the cox regression algorithm penalized by LASSO was utilized (Figure 4B, 4C). After cross validation, seven genes, including MT2A, PFKP, NMI, LATS2, CD74, PTPN6, CSF2RB, were chosen for calculation of a risk signature. Risk score of each patient was calculated with the gene expression value of seven genes and corresponding LASSO regression coefficient. The detailed formulation is: Risk=0.00164* MT2A expression + 0.00279*PFKP expression + 0.02065*NMI expression + 0.02099*LATS2 expression -0.00020*CD74 expression -0.00566*PTPN6 expression - 0.01433*CSF2RB expression. In addition, Kaplan Meier survival curve analysis using the log-rank method was carried out for these seven genes. The results showed that high expression of CD74 (p = 0.007), CSF2RB (p = 0.0096), or PTPN6 (p = 0.0083), showed the favorite prognosis, while high expression of MT2A (p = 0.0028), NMI (p < 0.0001), LATS2 (p = 0.0015), or PFKP (p = 0.0011) was associated with poor prognosis in LUAD (Figure 5).

We have also used the two-stage log-rank test to perform survival analysis with intersected curves for MT2A or CSF2RB gene. For MT2A gene, LRPV (p-value of the log-rank test) is 0.0028, MTPV (p-value of the suggested stage-II test) is 0.8000, and TSPV (p-value of the two-stage test) is 0.0028. In addition, for CSF2RB gene, LRPV is 0.0096, MTPV is 0.0000, and TSPV is 0.0096. Therefore, the conclusion is consistent with the previous one.

**Figure 6:**
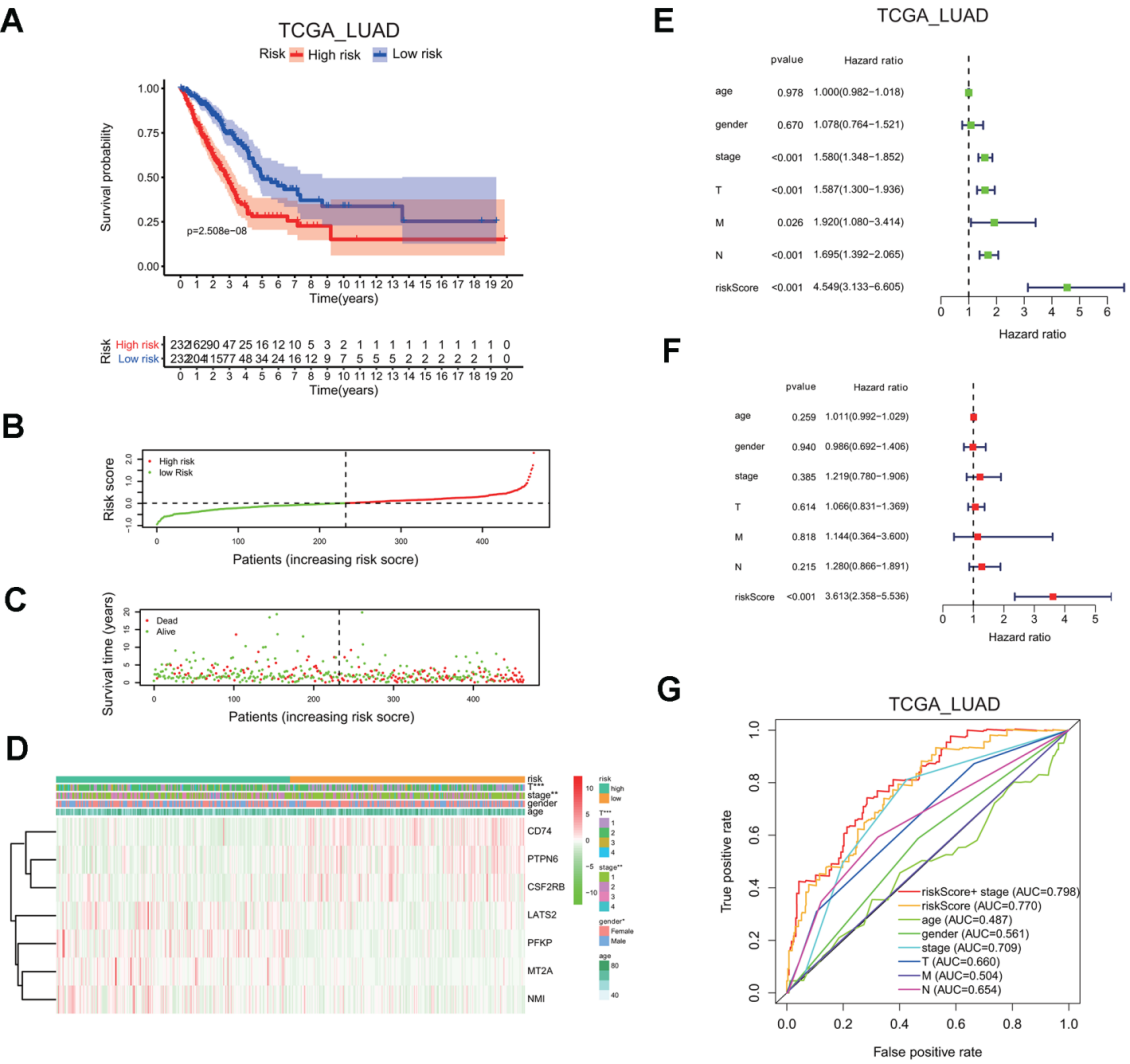
**Seven-gene risk signature can predict the prognosis in the TCGA LUAD cohort.** (**A**) Kaplan-Meier curves of overall survival probability of the high vs. low risk group. (**B**) Risk score distribution in the TCGA LUAD cohort. (**C**) Survival time and survival status distribution in the TCGA LUAD cohort. (**D**) Heatmap showing the expression level for seven interferon gamma response genes among the high or low risk group in the TCGA LUAD cohort. (**E**, **F**) Univariate (**E**) and multivariate (**F**) Cox regression analysis of the association between clinicopathological features, the risk score, and patient overall survival confirmed the signature as an independent factor of patient overall survival in the TCGA LUAD cohort. (**G**) ROC curves for 3-year survival prediction and clinical characteristics, including age, gender, stage (T, N, or M) in the TCGA LUAD cohort. AUC, area under curve.

LUAD patients in the TCGA cohort were classified into the low or high risk group, based on the median risk score of 0.1116, the overall survival probability in the high risk group is much lower than that in the low risk group (P=2.508e-8; Figure 6A). The risk score distribution and associated survival status also indicates higher risk score had more chances of death (Figure 6B, 6C). The expression levels of seven screened interferon gamma response genes in these two risk groups are presented in the heatmap (Figure 6D). LATS2, PEKP, MT2A, and NMI are upregulated in the high risk group, while CD74, PTPN6, CSF2RB are down-regulated in the high risk group. In addition, the correlation between the risk score and each clinical feature was analyzed, the late T stage (P<0.001), or stage (P<0.01) showed significant association with the high risk group (Figure 6D).

Meanwhile, HR of the risk score was 4.549 based on univariate Cox proportional hazards regression analysis (95% confidence interval (CI): 3.133–6.605; P<0.001; Figure 6E). Furthermore, multivariate Cox proportional hazards regression analysis revealed that HR of the risk score was 3.613 (95% CI: 2.358–5.536; P<0.001; Figure 6F). Finally, the ROC curve analysis showed that prognosis prediction had an area under the ROC curve (AUC) value of 0.770 when based on the risk score alone, or the AUC value of 0.798 when based on the combination of the risk score and the stage information (1 years; Figure 6G).

These findings indicated that the risk scores calculated based on the risk signature could accurately predict the prognosis and clinical features of the TCGA LUAD cohort.

### Validation of the risk signature using an independent cohort from the GEO database

**Figure 7:**
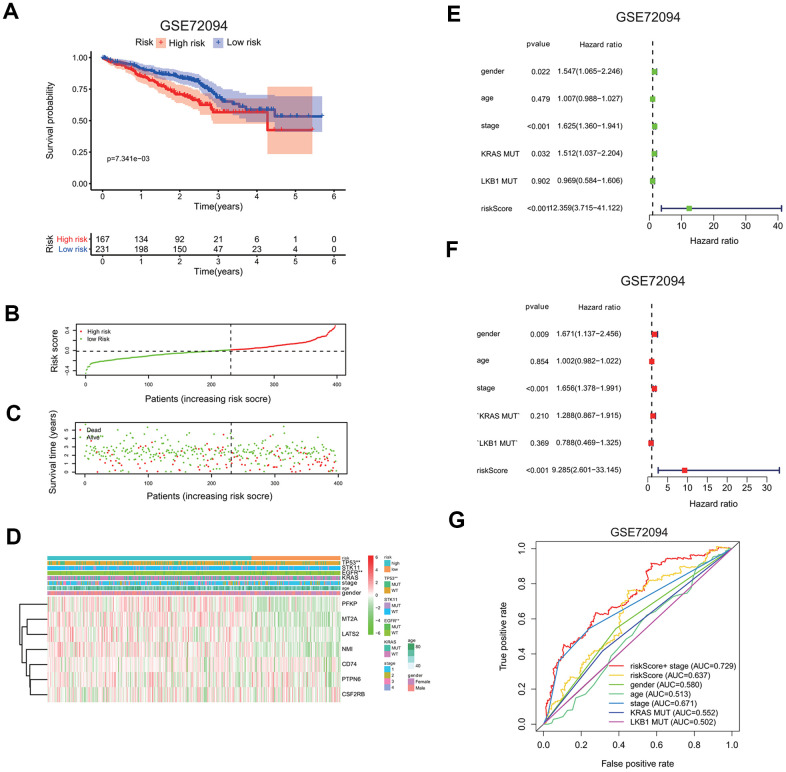
**Validation of seven-gene risk signature in an independent LUAD cohort.** (**A**) Kaplan-Meier curves of overall survival of the high or low risk group in the GSE72094 cohort. (**B**) Risk score distribution in the GSE72094 cohort. (**C**) Survival time and survival status distribution in the GSE72094 cohort. (**D**) Heatmap showing the expression level of seven interferon gamma response genes in the low or high risk group in the GSE72094 cohort. Association of various clinical features with two groups (high vs. low risk) were determined. *P<0.05, **P<0.01 and ***P<0.001. (**E**, **F**) Univariate (**E**) and multivariate (**F**) Cox regression analysis of the association between clinical features (gender, age, stage, KRAS or LKB1 mutation), risk score, and patient overall survival in the GSE72094 cohort. Hazard ratio and associated p value are shown. (**G**) ROC curves for 3-year survival prediction and clinical characteristics, including age, gender, stage, KRAS or LKB1 mutation in the GSE72094 cohort.

To confirm the risk signature independently, we applied seven-gene prognostic signature to an independent lung cancer cohorts in the GEO database (GSE72094). The clinicopathological information of GSE72094 patients was summarized here (Supplementary Table 1B). The high risk group had a markedly reduced survival probability than the low risk group (GSE72094: p=7.341e-3; Figure 7A). The increase in the risk scores of patients is correlated with more death in patients (Figure 7B, 7C), implying the higher risk score carries more chance of death for patients.

The expression levels of seven genes were also compared between the high and low risk group, as shown in the heatmaps (Figure 7D). PFKP, MT2A, LATS2, and NMI expressions were increased, while CD74, PTPN6, CSF2RB expressions are decreased in the high risk group in the GSE72094 cohort. Interestingly, TP53 or EGFR mutation is significantly associated with the high risk group.

Univariate and multivariate Cox regression analysis were carried out to evaluate whether the risk score can serve as an independent prognostic factor compared to other clinical features, including gender, age, gene mutations, or stage. In univariate cox regression analysis, for GSE72094, HR of the risk score is 12.359 (95% CI: 3.715 to 41.122) with p <0.001 (Figure 7E). In multivariate cox analysis, for GSE72094, HR of the risk score is 8.297 (95% CI: 3.715 to 41.122) with p <0.001 (Figure 7F); This analysis indicates that the risk signature could serve as independent prognostic factors in this lung cancer data.

The ROC curve analysis was done to assess the prognostic accuracy of the risk signature. For GSE72094, the prognostic prediction has the AUC value of 0.637 based on the risk score, which was higher than other clinical features, including gender (0.580), age (0.513), KRAS mutation (0.552), LKB1 mutation (0.502), but was a little lower than stage (0.637). The AUC value increased to 0.729 when prognostic predication was assessed by the combination of both the risk score and stage information (3 years; Figure 7G).

Nomogram of TCGA LUAD and GSE72094 cohorts were also presented to quantify the whole risk score for seven genes based on the clinical features (Supplementary Figure 3A, 3B).

### Functional analysis of genes associated with the high and low risk group

Genes associated with the high and low risk group of LUAD patients from the TCGA cohort were analyzed by GSEA for enrichment analysis of KEGG pathways, in order to examine the potential biological functions in each group. Genes associated with the high risk group are enriched in pathways including proteasome, cell cycle, DNA replication, spliceosome, DNA mismatch repair, etc, while genes associated with the low risk group are enriched in pathways including thyroid disease, allograft rejection, intestinal immune network for IGA production, or graft versus host disease, which are related to immune function (Supplementary Figure 4A).

We carried out similar analysis for the GSE72094 cohort, genes in the high risk group were enriched in pathways including mismatch repair, base excision repair, cell cycle, pyrimidine metabolism, and p53 signaling pathway, etc, and genes in the low risk group were enriched in pathways including GnRH signaling pathway, Fc epsilon RI signaling pathway, valine, leucine and isoleucine degradation, other glycan degradation, etc (Supplementary Figure 4B).

Overall, genes in the high risk group were more likely enriched in pathways including cell cycle, DNA replication, mismatch repair pathways, etc, while genes in the low risk group were more likely enriched in immune function related pathways. Thus, this analysis uncovers the underlying difference in cellular pathways in the high and low risk groups.

### The risk signature defines distinct immune response, immune cell infiltration, and immune checkpoint gene expression in lung adenocarcinoma

In order to further investigate the underlying connection between risk groups and immune function or cell signaling pathways in lung cancer, GSVA analysis was used to calculate the enrichment levels of different gene sets for each sample.

**Figure 8:**
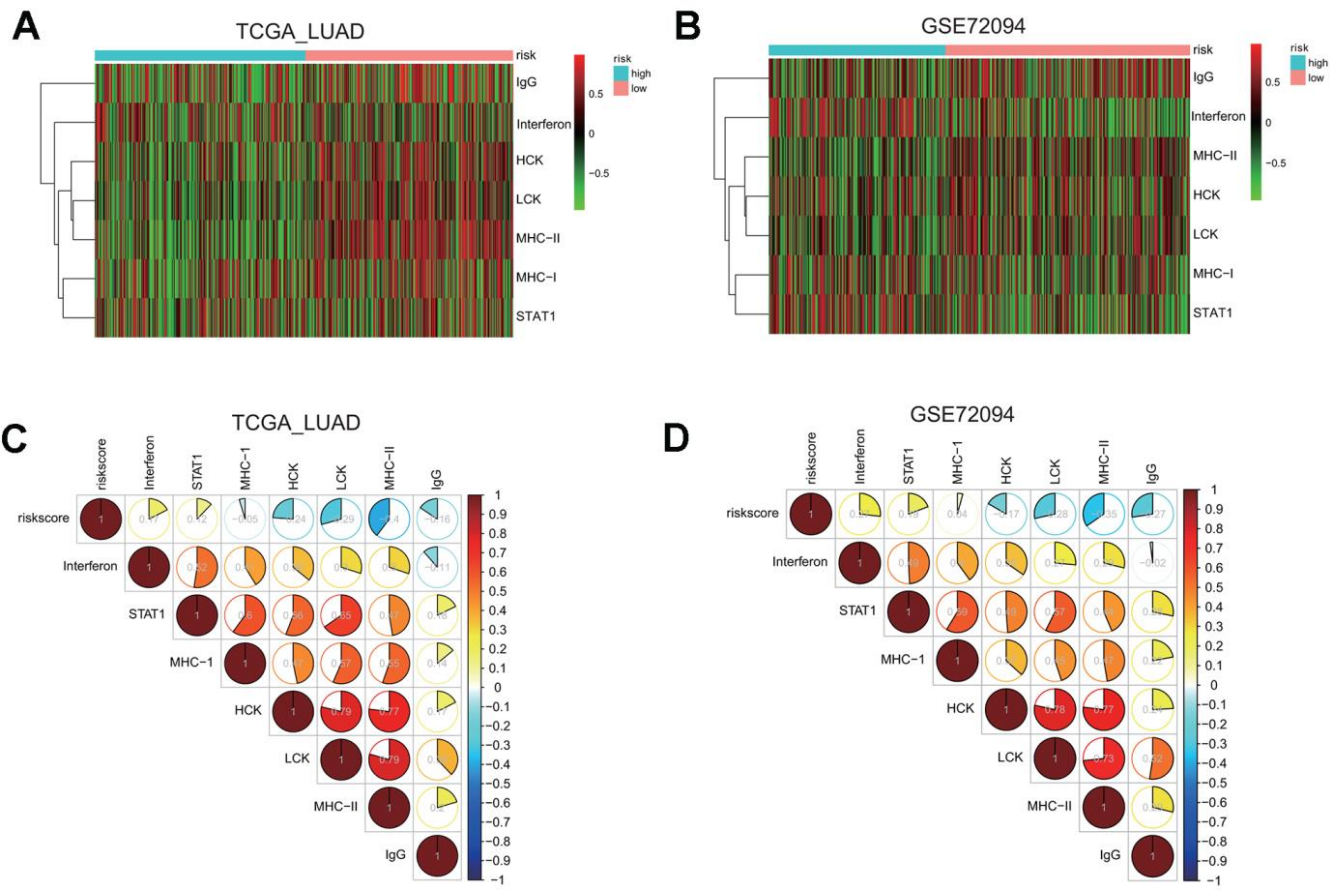
**Gene set variation analysis (GSVA) and correlation coefficient analysis between immune-related metagenes and the risk score in two LUAD cohorts.** (**A**, **B**) Heatmaps of the enrichment levels of immune-related metagenes, including IgG, Interferon, HCK, LCK, MHC-I, MHC-II, and STAT1, in the high or low risk group in the LUAD cohorts: TCGA (**A**) and GSE72094 (**B**). (**C**, **D**) Correlograms of the risk score and seven immune-related metagenes in the LUAD cohorts: TCGA (**C**) and GSE72094 (**D**).

First, we examined the association between risk groups and seven immune metagenes, including interferon, STAT1, MHC-I, HCK, LCK, MHC-II, and IgG, representing different aspects of immune response [25]. GSVA results for the TCGA and GSE72094 cohorts are presented in the heatmap (Figure 8A, 8B), revealing significant difference in the enrichment level of these metagenes between the high and low risk groups.

To quantify their associations, the correlogram was used to display correlation between the risk score, and seven immune metagenes, based on the Pearson’s correlation coefficient between the risk score and seven metagenes (Figure 8C). The analysis indicated that the risk score was negatively correlated with HCK, LCK, MHC-II, or IgG metagenes in the TCGA LUAD cohort. To validate these findings, GSVA were also carried out in the GSE72094 cohort, and showed that the risk score was negative correlated with HCK, LCK, MHC-II, or IgG metagenes as well (Figure 8D), indicating that the risk score derived from our prognostic gene signature was negatively associated with immune activities including antigen presentation and T cell activation.

We also extended the GSVA analysis of each sample in these two LUAD cohorts to several KEGG biological pathways, and found that the high risk group were enriched with base excision repair, mismatch repair, or nucleotide excision repair pathway in all two LUAD cohorts examined (Supplementary Figure 5A, 5B). The correlogram confirmed that the risk score is positively correlated with three DNA damage repair pathways, including base excision repair, nucleotide excision repair, and mismatch repair, but negatively correlated with PPAR signaling, cell adhesion molecules, and basal cell carcinoma related pathways (Supplementary Figure 5C, 5D).

**Figure 9:**
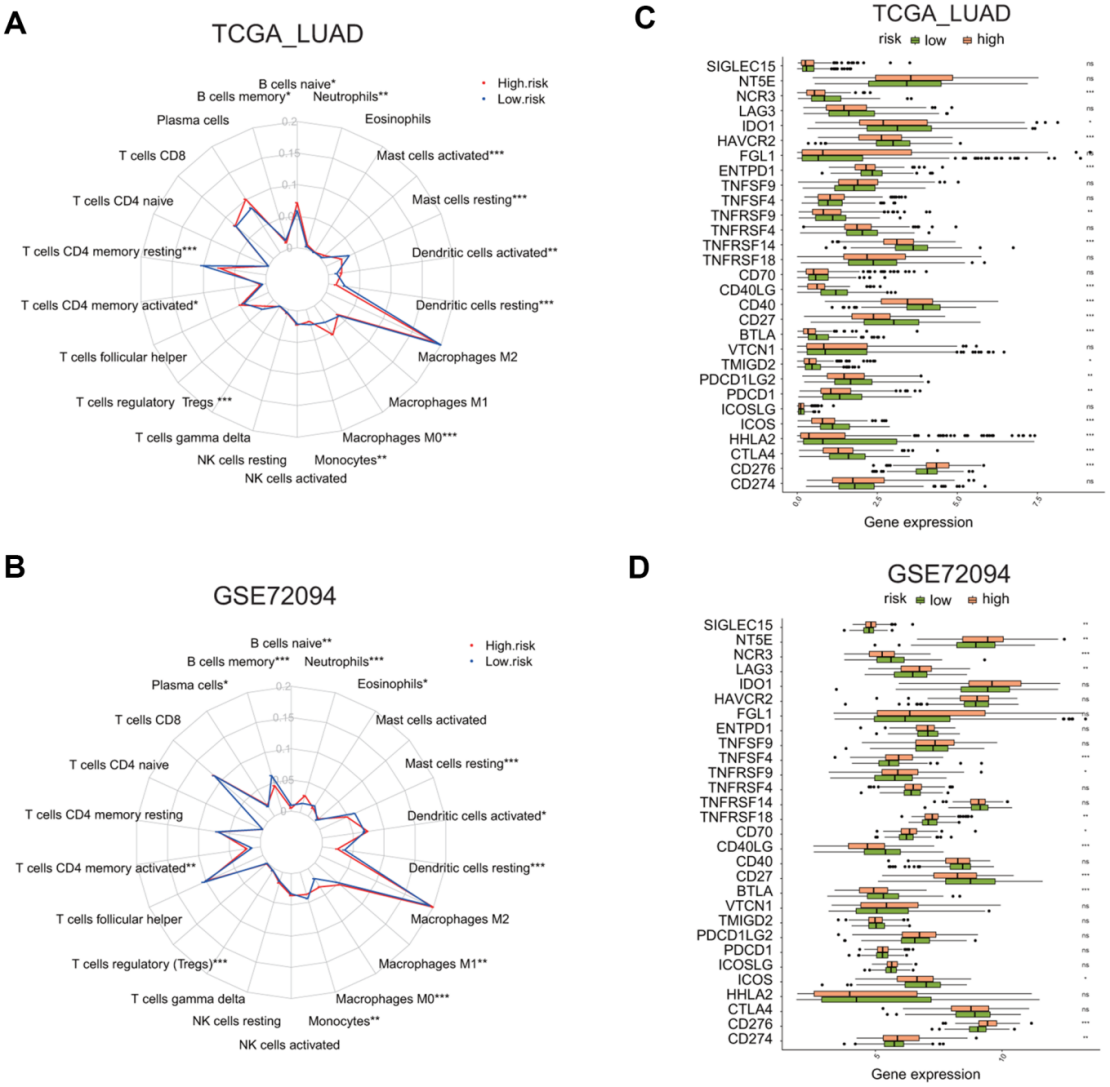
**Comparison of immune cell infiltration and immune checkpoint gene expression between the high and low risk groups in two LUAD cohorts.** (**A**, **B**) Radar plots show the distribution of the enrichment levels of 22 immune cells in the high (red line) or low (blue line) risk group in the LUAD cohorts: TCGA (**A**) and GSE72094 (**B**). Statistically significant difference in the enrichment level of immune cells between the high and low risk groups is indicated by the asterisks. *P < 0.05, **P < 0.01, ***P < 0.001. (**C**, **D**) Bar plots show the comparison of the expression of 29 immune checkpoint genes between the low and high risk groups in two LUAD cohorts: TCGA (**C**) and GSE72094 (**D**), by *P < 0.05, **P < 0.01, ***P < 0.001.

Next, we asked whether two risk groups have different immune cells infiltration profile by using CIBERSORT analysis. The relative enrichment levels of 22 immune cells were shown in the radar plots (Figure 9A, 9B). Wilcox test was carried out to determine the difference in the enrichment level of immune cells between the high or low risk groups. In the TCGA LUAD cohort, the high risk group had the markedly higher levels of naïve B cells, activated memory CD4 T cells, macrophage M0 cells, activated dendritic cells, and neutrophils, but the lower level of memory B cells, resting memory CD4 T cells, regulatory T cells, resting dendritic cells, and resting mast cells (Supplementary Figure 6A).

In the GSE72094 LUAD cohort, the high risk group had the markedly higher level of plasma cells, activated memory CD4 T cells, macrophage M0 and M1 cells, activated dendritic cells, and neutrophils, but the lower level of memory B cells, monocytes, resting dendritic cells, resting mast cells, eosinophils (Supplementary Figure 6B).

We also analyzed the differential expression of 29 immune checkpoint molecules or related TNF superfamily members, including CD274, CD276, CTLA4, HHLA2, ICOS, ICOSLG, PDCD1, PDCD1LG2, TMIGD2, VTCN1, BTLA, CD27, CD40, CD40LG, CD70, TNFRSF18, TNFRSF14, TNFRSF4, TNFRSF9, TNFSF4, TNFSF9, ENTPD1, FGL1, HAVCR2, IDO1, LAG3, NCR3, NT5E, SIGLEC15 [26–29] (Figure 9C, 9D). The analysis revealed that, in the TCGA LUAD cohort, the high risk group has a higher expression of CD276, but the reduced expression of CTLA4, HHLA2, ICOS, PDCD1, PDCD1LG2, TMIGD2, BTLA, CD27, CD40, CD40LG, TNFRSF14, TNFRSF9, ENTPD1, HAVCR2, IDO1, or NCR3 (Supplementary Figure 7A, p < 0.05). In the GSE72094 LUAD cohort, the high risk group has a higher expression of CD274, CD276, CD70, TNFRSF18, TNFRSF9, TNFSF4, LAG3, NT5E, or SIGLEC15, but reduced expression of ICOS, BTLA, CD27, CD40LG, or NCR3 (Supplementary Figure 7B, p < 0.05). This analysis indicates that the high or low risk group has distinctive immune checkpoint gene expression profiles.

Consistent with the above analysis, we also found that, in the TCGA LUAD cohort, the high risk group has higher tumor purity, higher tumor mutation burden (TMB), but lower stromal score, lower immune score (Supplementary Figure 8A–8C, P < 0.05).

## DISCUSSION

The present study examined the expression, mutation pattern, and protein-protein interaction of 24 interferon gamma response genes in lung adenocarcinoma. To screen for interferon gamma response genes with prognostic value, first, consensus clustering of lung cancer patients was carried out based on 24 interferon gamma response gene expression. two LUAD subgroups, namely cluster 1 and 2, were identified. Specifically, the cluster 2 subgroup has significantly lower survival rate and exhibited a close correlation with late T stage. Second, univariate cox regression analysis was carried out to determine the genes with significant HR values for survival analysis; third, LASSO-penalized cox regression was used to enhance the accuracy of our prognosis model to identifying the most significant variables. A risk signature for prognosis was generated based on seven interferon gamma response genes, which can divide LUAD patients into the low or high risk group. Finally, the risk signature was successfully validated in an independent GEO dataset, and could serve as an independent predictor for lung cancer prognosis, indicating the general applicability of this risk signature.

The benefits of these approaches are discussed here. Consensus clustering is a powerful unsupervised machine learning algorithm, which can produce a combined clustering unattainable by any single clustering method and are less sensitive to noise, outliers or sample variations. It can be used to mine clinically relevant and previously unknown disease classifications in gene expression data. Next, cox regression analysis is method for investigating the effect of several variables upon the time a specified event happening. It generates a better estimate of these functions than the Kaplan-Meier method when the assumptions of the Cox model are met and the fit of the model is strong. In addition, LASSO-penalized cox regression produces simpler and more interpretable models that incorporate only a reduced set of the predictors.

Interestingly, both MT2A and LATS2 were significantly down-regulated in the lung cancer samples compared to the normal control in the TCGA LUAD cohort (Figure 1B), but the univariate cox analysis of overall survival revealed that both MT2A and LATS2 were the risk genes with a HR of more than 1 (Figure 4), which was validated by our Kaplan Meier survival curve analysis (Figure 5). We validated the accuracy of our analysis by getting the same results using the web-based analysis tool (http://gepia.cancer-pku.cn/index.html). The discrepancy may be due to several reasons, such as: different splicing forms of MT2A or LATS2 proteins may have different roles in tumor development; different lengths of patient follow-up used in the survival analysis may yield different HRs, etc.

Among these seven genes, MT2A acts as anti-oxidant, protects cells against hydroxyl free radicals, is important in homeostatic control of metal in the cell. HMBOX1 interacts with MT2A to regulate intracellular free zinc level, to inhibit apoptosis and promote autophagy in VECs [30]. PFKP encodes a member of the phosphofructokinase. A protein family, plays a key role in glycolysis regulation. PFKP is up-regulated in lung cancer and regulates glucose metabolism [31]. It is a prognostic marker in breast cancer [32]. LATS2 encodes a serine/threonine protein kinase. It can phosphorylate and stabilize SNAI1 in the nucleus, which promotes epithelial-mesenchymal transition and tumor cell invasion. LATS2 is as a poor prognostic marker in non-small cell lung cancer [33]. NMI interacts with CMYC, NMYC, or STATs, and enhances STAT-mediated transcription upon cytokines IL2 and IFN-gamma stimulation. NMI promotes cell proliferation through TGFbeta/Smad pathway by upregulating STAT1 in colorectal cancer [34]. CD74 binds class II major histocompatibility complex (MHC) and serves as a chaperone regulating antigen presentation during immune response [35]. It also binds the cytokine macrophage migration inhibitory factor (MIF) to promote cell survival and proliferation [36]. PTPN6 encodes a member of the protein tyrosine phosphatase (PTP) family, which regulates multiple cellular processes, such as cell growth, differentiation, and tumorigenesis [37]. CSF2RB is the common beta chain of the high affinity receptor for IL-3, IL-5 and CSF. Defects in CSF2RB is associated with a lung condition called protein alveolar proteinosis (PAP) [38].

Using the as-constructed risk signature, LUAD patients from two different cohorts can be divided into the high and low risk group, and the high risk group showed significantly poorer survival rate than the low risk group. GSEA analysis revealed that genes in the high risk group are associated with biological processes, such as, cell cycle, DNA replication, base excision repair, and mismatch repair, etc, while genes in the low risk group are enrichment in immune related pathways, such as, allograft rejection, intestinal immune network for IGA production, or graft versus host disease, etc. Furthermore, GSVA analysis indicates the high risk group is negatively correlated with several immune profiling signature (including interferon, LCK, HCK, etc), but highly correlated with DNA repair pathways, including DNA mismatch repair, nuclear excision repair, etc. This result indicates that DNA repair pathway may be involved in negatively regulating immune pathways in lung cancer. Finally, the high risk group has higher tumor purity, and higher tumor burden, but lower stroma score, immune score, and estimate score, which is consistent with our previous analysis.

Immune cell infiltration in tumor microenvironment is an important regulator of tumor progression, our analysis found that there was a significant difference in immune cell infiltration between the high and low risk group of lung cancer patients in three different cohorts. The high risk group has the higher enrichment of immune cells including macrophage M0 cells, activated dendritic cells, activated memory CD4 T cells; while the low risk group has higher enrichment of immune cells including memory B cells, resting memory CD4 T cells, resting dendritic cells, resting mast cells, monocytes, etc. This analysis indicates that the high risk score may represent an immunosuppressive microenvironment.

In addition, several immune checkpoint genes were also differentially expressed in the high and low risk group of lung cancer patients in the cohorts, the high risk group has the higher expression of CD276 in both TCGA and GSE72094 LUAD cohorts, while the low risk group has the higher expression of CD27, CD40LG, or NCR3 in both cohorts, indicating the possible use of blockers targeting different immune checkpoint genes for different risk groups.

In conclusion, our study comprehensively illustrated the expression and mutation patterns, possible role and prognostic significance of important interferon gamma response genes in lung adenocarcinoma patients, and derived a risk signature composed of seven genes, which could accurately predict the prognosis in lung adenocarcinoma patients. The present study will contribute to enhancing our understanding of interferon gamma response genes, its role in regulating cellular pathways, affecting immune cell-infiltration and immune checkpoint gene expression in tumor microenvironment. This study may help to guide more effective immunotherapy strategies again lung cancer.

## MATERIALS AND METHODS

### Data acquisition

Gene expression data, somatic mutation data, and clinical data of LUAD patients were downloaded from the TCGA dataset (https://tcga-data.nci.nih.gov/) using the GDC Data Transfer Tool. A total of 551 LUAD patient samples (497 tumor and 54 normal samples) were used in this study to identify the differentially expressed interferon gamma response genes. The GSE72094 dataset was acquired from the GEO database (https://www.ncbi.nlm.nih.gov/geo/). The clinicopathological information of two LUAD cohorts were summarized (Supplementary Table 1). For TCGA LUAD cohort, only 476 samples without missing clinical features were included in the Supplementary Table 1.

### Bioinformatic analysis

Difference comparison between two groups (for example, gene expression in tumor vs. normal samples) was carried out using Wilcoxon tests, with p value < 0.05 as statistically significant. Difference comparisons of three or more groups were carried out using One-way ANOVA and Kruskal-Wallis tests. Correlation coefficients between genes were calculated by Spearman correlation coefficient analysis using the ‘corrplot’ R package. In addition, the interactions between interferon gamma response genes/proteins were obtained from the STRING website (http://www.string-db.org/).

LUAD were clustered in various groups based on the expression pattern of interferon gamma response genes using the ‘ConsensusClusterPlus’ R package. Principal component analysis (PCA) analysis of LUAD samples were investigated using the ‘FactoMineR’ and ‘factoextra’ R packages. The mutation landscape in the TCGA LUAD cohort was presented by the ‘maftools’ R package. The ‘estimate’ R package calculates stromal and immune scores that represent the presence of stromal and immune cells in tumor tissue, respectively. Kaplan Meier survival curve analysis was carried out using the ‘survival’ R package, the optimal cutpoint value for each gene was determined by the surv_cutpoint function in the ‘survminer’ R package. Two-stage log-rank test in the ‘TSHRC’ R package was used to perform the survival analysis with crossed curves.

All statistical P values were two-tailed, and were statistically significant when p < 0.05. All data analysis was conducted in R software (version 3 .6.1).

### Construction of a signature based on interferon gamma response genes

A univariate Cox regression model was adopted to determine the hazard ratios (HR) of prognosis prediction for interferon gamma response genes. Univariate or multivariate Cox analysis was employed to determine the prognostic value for the risk signature or clinical features using the ‘forest’ R package.

Genes were further screened by Least Absolute Shrinkage and Selection Operator (LASSO) regression analysis, followed by 10-fold cross validation using the ‘glmnet’ R package. Seven genes with their regression coefficient (Coef) were selected, the risk score for each patient was calculated through linearly multiplying the expression level with Coef of each gene, according to the following formula: Risk score =Coef _gene1_ × expression _gene1_ + Coef _gene2_ × expression _gene2_ + · ···· + Coef _gene n_ × expression _gene n_.

### Confirmation of the signature based on interferon gamma response genes

All patients in the LUAD cohorts were separated into the high or low risk group based on the median risk score. Survival curves were drawn based on the risk score using the Kaplan-Meier method. Student’s t-test and Pearson’s test were used to compare the significant association between risk scores and various clinical features.

Subsequently, the receiver operating characteristic (ROC) curves were performed to assess the sensitivity and specificity of survival prediction by the risk signature using the ‘survivalROC’ R package. Univariate and multivariate Cox proportional hazards regression analysis was performed to determine whether the risk score is an independent predictor for prognosis.

### Gene set enrichment analysis (GSEA) and gene set variation analysis (GSVA)

GSEA for the mRNAs associated with the high or low risk group was carried out using c2.cp.kegg.v7.1.symbols.gmt as gene sets database at 1,000 random sample permutations using JAVA application (http://software.broadinstitute.org/gsea/index.jsp).

To investigate the difference on biological pathways between high and low risk groups, GSVA enrichment analysis was carried out using the “GSVA” R package. GSVA, a non-parametric and unsupervised method, is used for calculates sample-wise gene set enrichment scores [39]. The gene sets for pathways are in the “c2.cp.kegg.v7.1.symbols” document from the MSigDB database.

### Immune cell infiltration estimation

The CIBERSORT method was used to calculate the enrichment levels of immune cell infiltration in lung cancer cohorts. The difference in the immune cell infiltration between the low and high risk groups was carried out using Wilcoxon tests, with the p value < 0.05 as statistically significant.

The gene set for each immune cell signature, as well as various pro- or anti-tumor immune cell subtypes, came from this study [40]. The enrichment scores determined by CIBERSORT were used to represent the relative abundance of each immune cell in LUAD samples.

### Data availability statement

The data that support the findings of this study are openly available from the TCGA dataset (https://tcga-data.nci.nih.gov/). The GSE72094 dataset is available from the GEO database (https://www.ncbi.nlm.nih.gov/geo/).

## Supplementary Material

Supplementary Figures

Supplementary Tables
