# Supplementary material for: Seven interferon gamma response genes serve as a prognostic risk signature that correlates with immune infiltration in lung adenocarcinoma
Source: Aging (Albany NY). 2021 Apr 4;13(8):11381–410. doi: 10.18632/aging.202831 (PMC8109098; doi:10.18632/aging.202831)
Supplement: Supplementary Figures [file aging-13-202831-s001.pdf]

SUPPLEMENTARY FIGURES

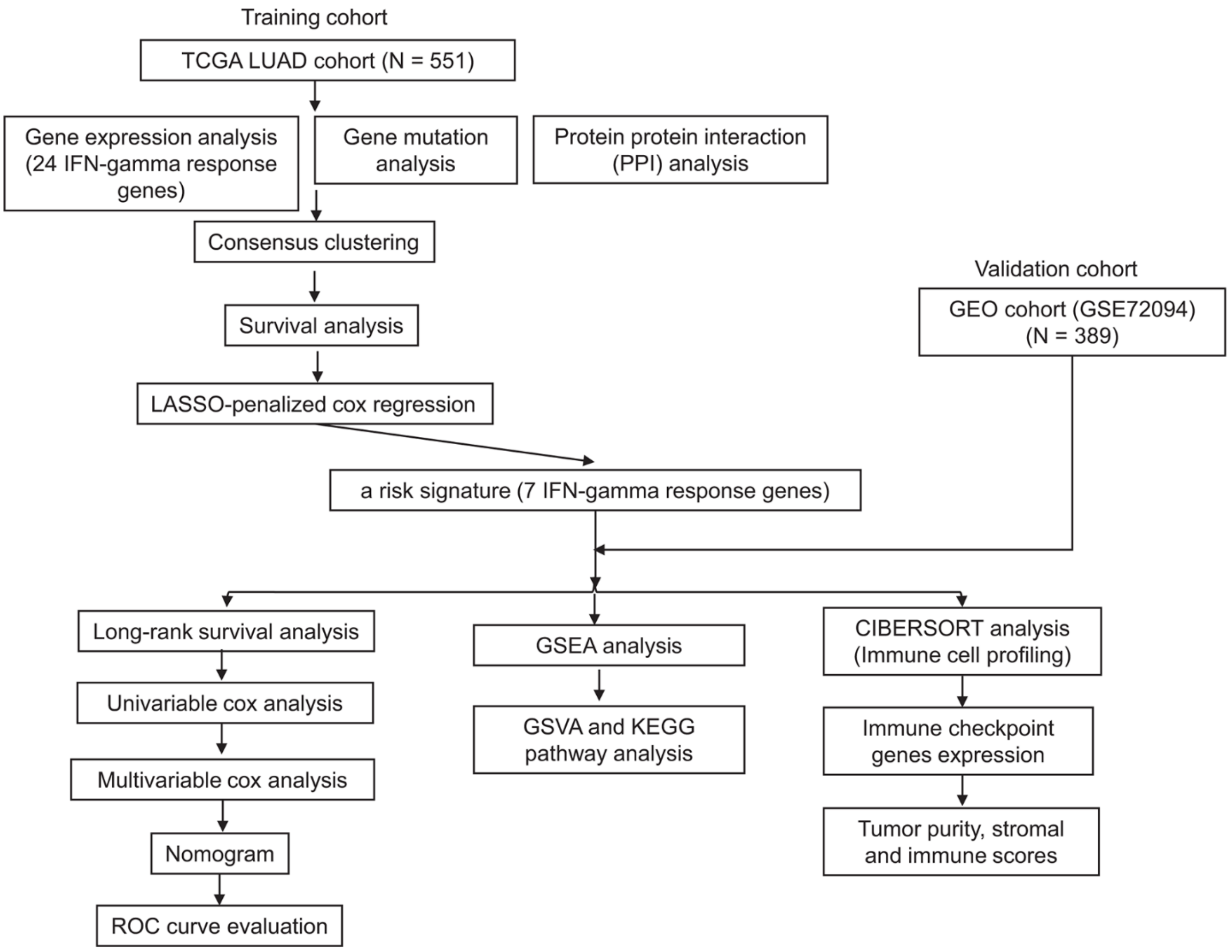

Supplementary Figure 1. Flow chart of the study.

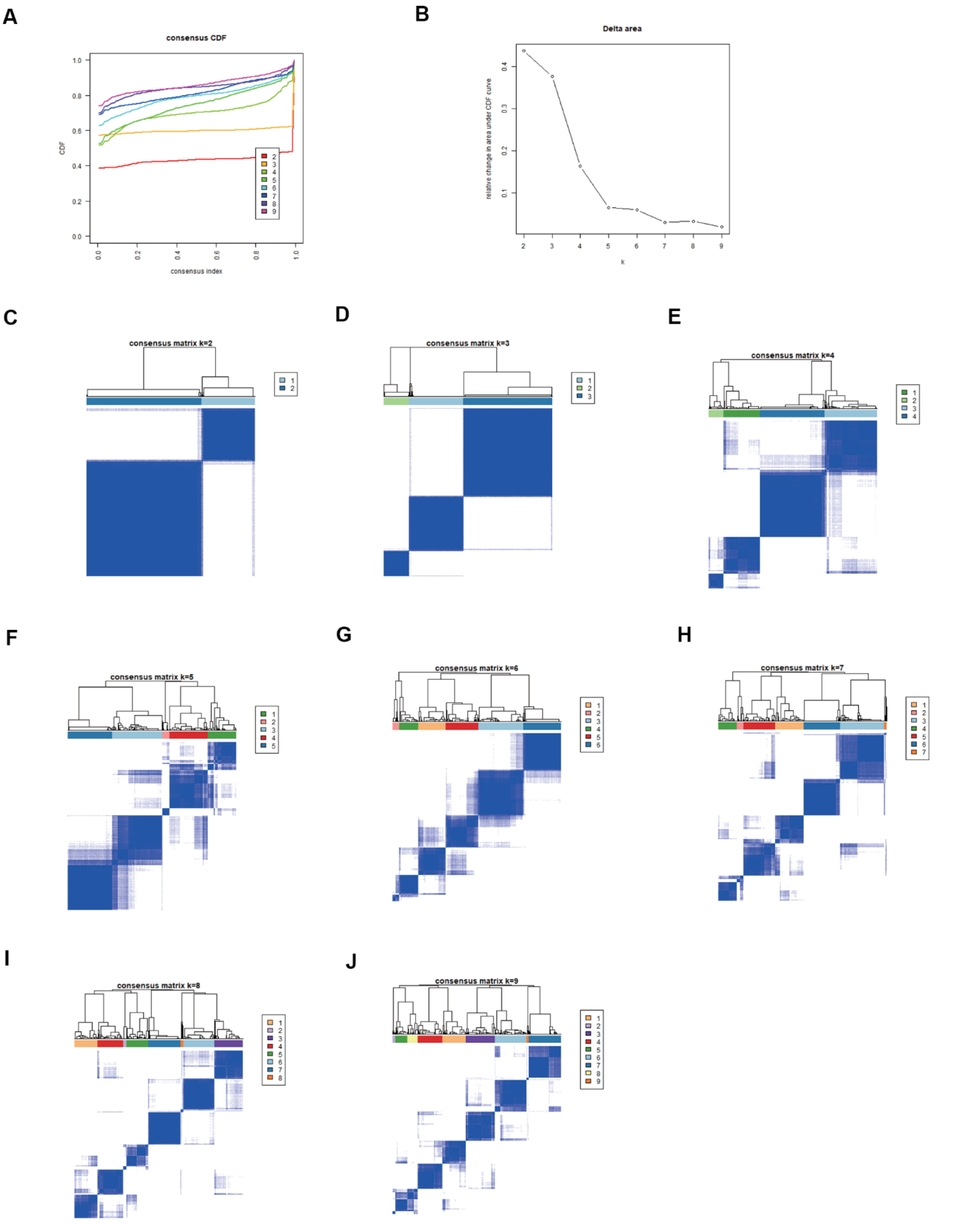

Supplementary Figure 2. Consensus clustering of 24 interferon gamma response genes identified two clusters of LUAD in the TCGA LUAD cohort. (**A**) Cumulative distribution function (CDF) plot displays consensus distributions at k=2 to k=9. (**B**) Relative changes in the area under the CDF curve at k=2 to k=9. (**C–J**) Consensus matrix display at k=2 (**C**) to k=9 (**J**).

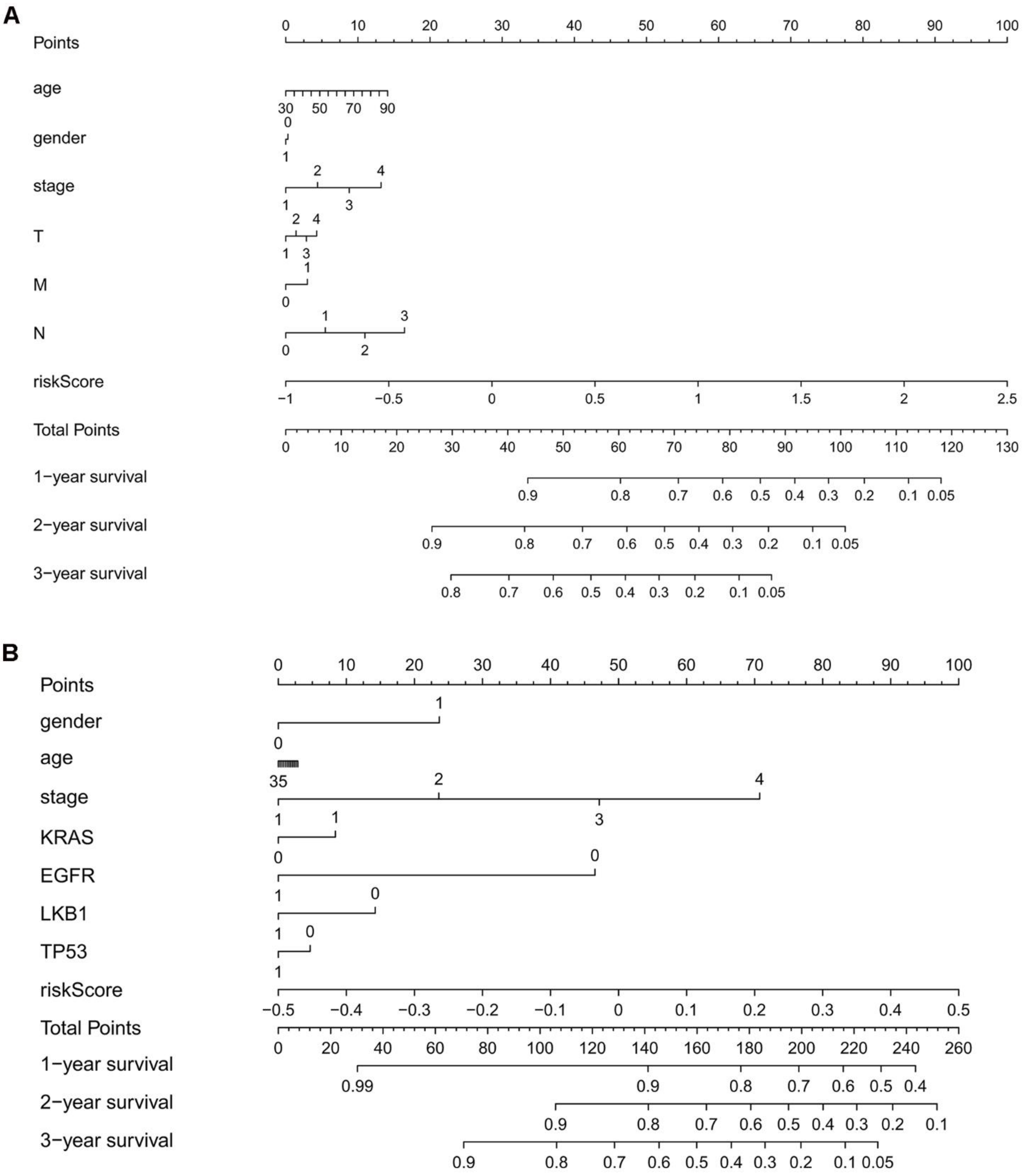

Supplementary Figure 3. Nomogram of TCGA LUAD and GSE72094 cohorts to quantify the whole risk score for seven genes based on the clinical features. (**A**) LUAD cohort. (**B**) GSE72094 cohort.

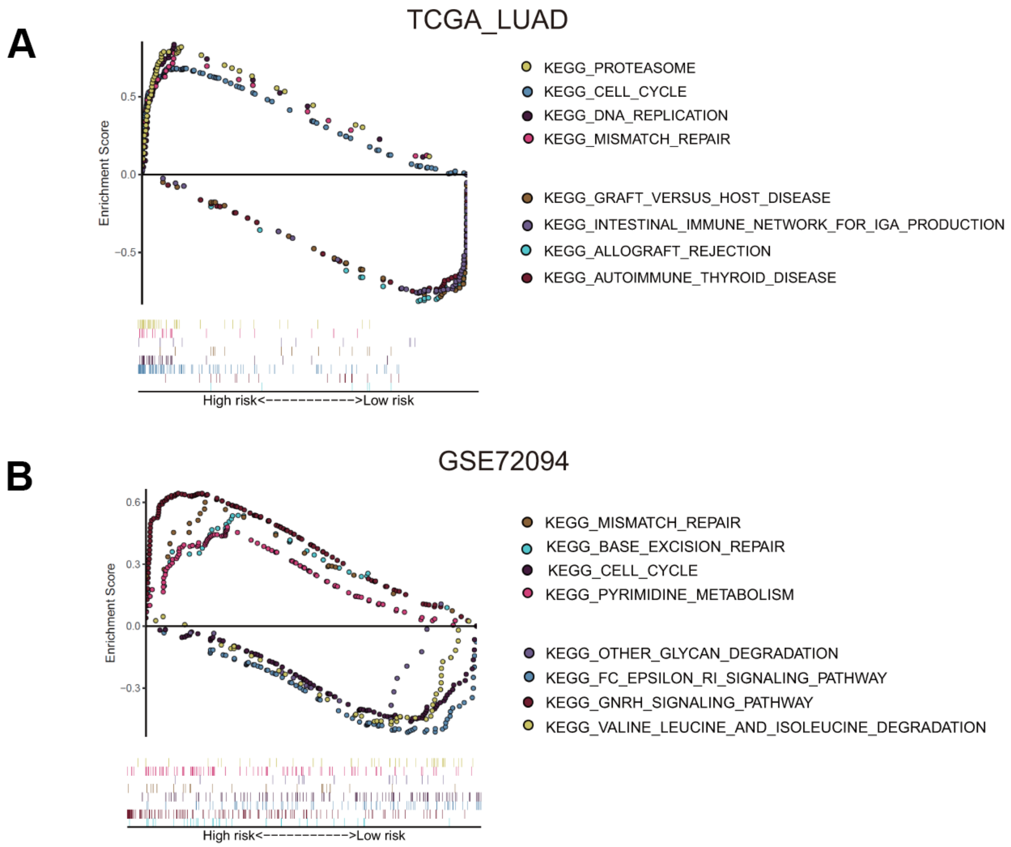

Supplementary Figure 4. Gene set enrichment analysis (GSEA) of the mRNAs associated with the high or low risk group in two LUAD cohorts. (**A**, **B**) Top enriched KEGG pathways in the high risk group are represented by the curves above the x-axis in the graph. Top enriched KEGG pathways in the low risk group are represented by the curves below the x-axis in the graph (p-value < 0.05) for two LUAD cohorts: TCGA (**A**) and GSE72094 (**B**). The names of enriched KEGG pathways are listed on the right side.

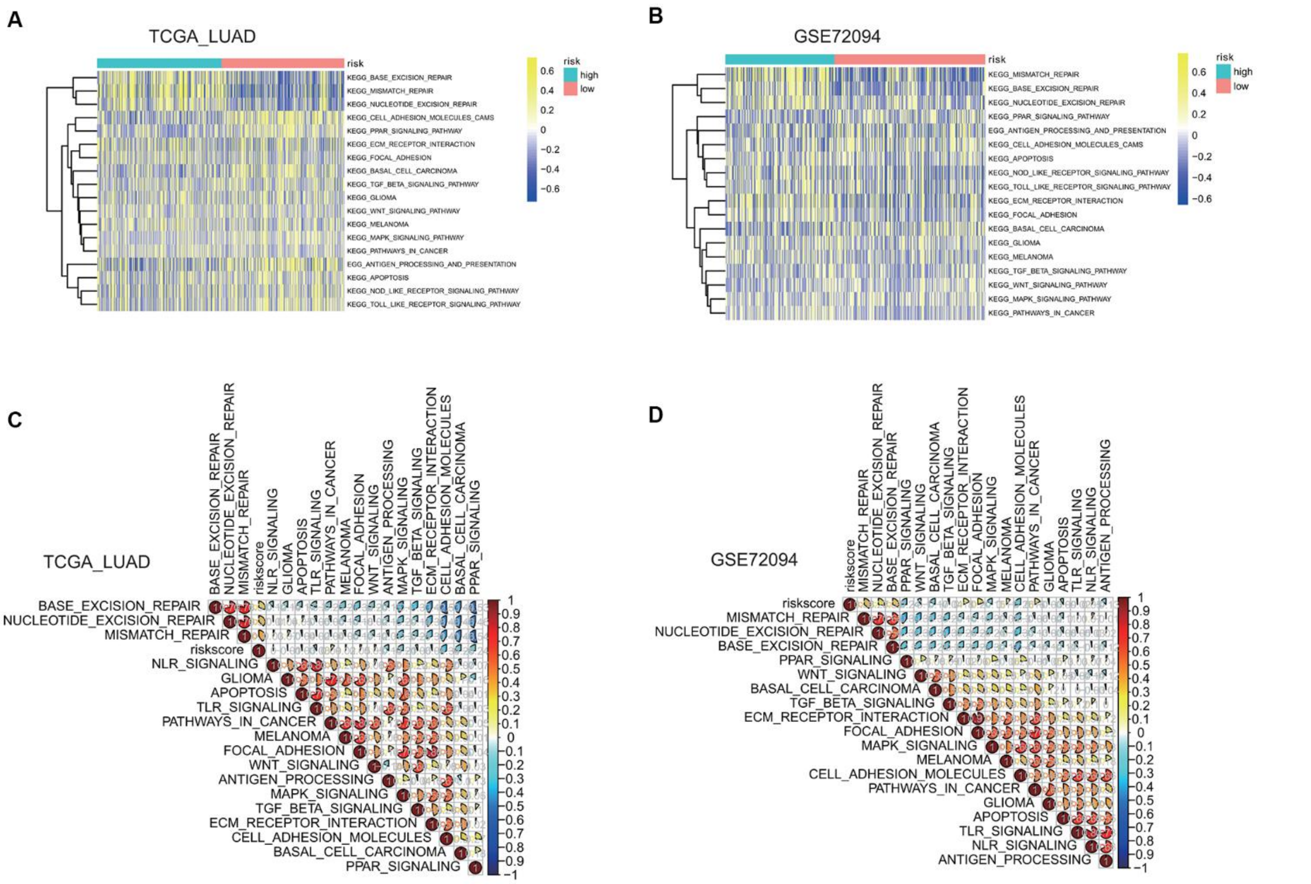

Supplementary Figure 5. GSVA and correlogram analysis of KEGG biological pathways based on risk groups or risk scores in two LUAD cohorts. (**A**, **B**) Heatmaps visualizing the enrichment levels of KEGG biological pathways in the high or low risk group in the LUAD cohorts: TCGA (**A**) and GSE72094 (**B**). Yellow represents enriched pathways, and blue represents repressed pathways. (**C**, **D**) Correlograms demonstrating the correlation coefficient between the risk score and KEGG pathways in the LUAD cohorts: TCGA (**C**) and GSE72094 (**D**). Red color depicts the positive correlation, and blue color depicts the negative correlation.

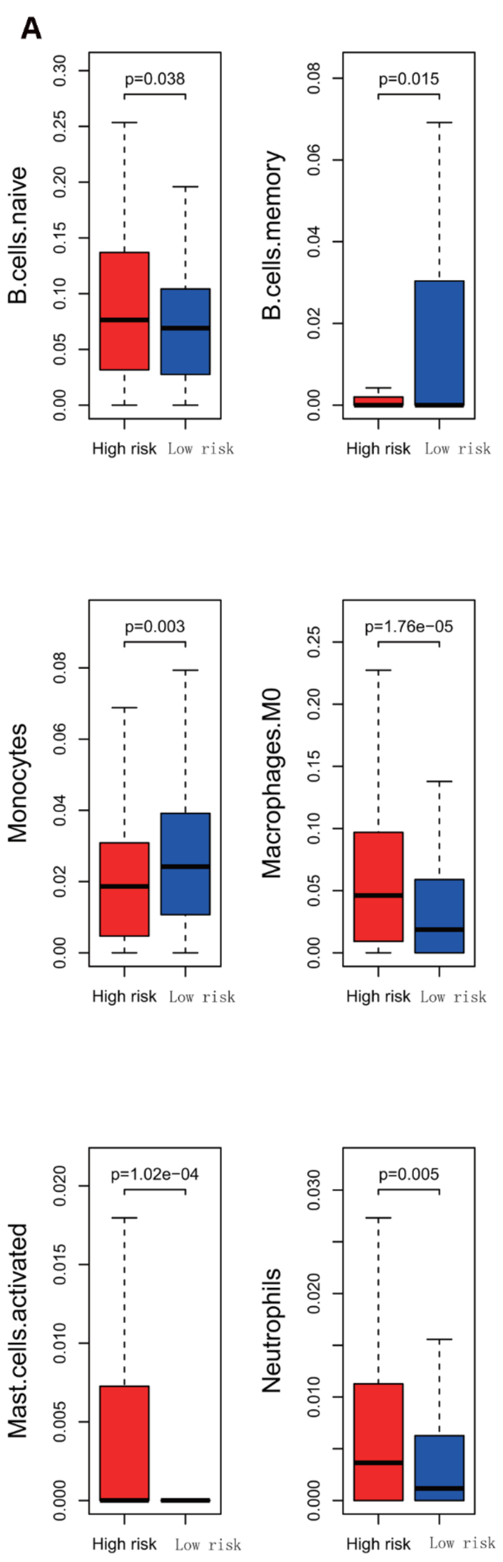
TCGA_LUAD
A
B.cells.naive
p=0.038
B.cells.memory
p=0.015
T.cells.CD4.memory.resting
p=1.071e−06
T.cells.CD4.memory.activated
p=0.020
T.cells.regulatory..Tregs.
p=7.3e−05
Monocytes
p=0.003
Macrophages.M0
p=1.76e−05
Dendritic.cells.resting
p=8.309e−11
Dendritic.cells.activated
p=0.007
Mast.cells.resting
p=3.694e−07
Mast.cells.activated
p=1.02e−04
Neutrophils
p=0.005
High risk
Low risk

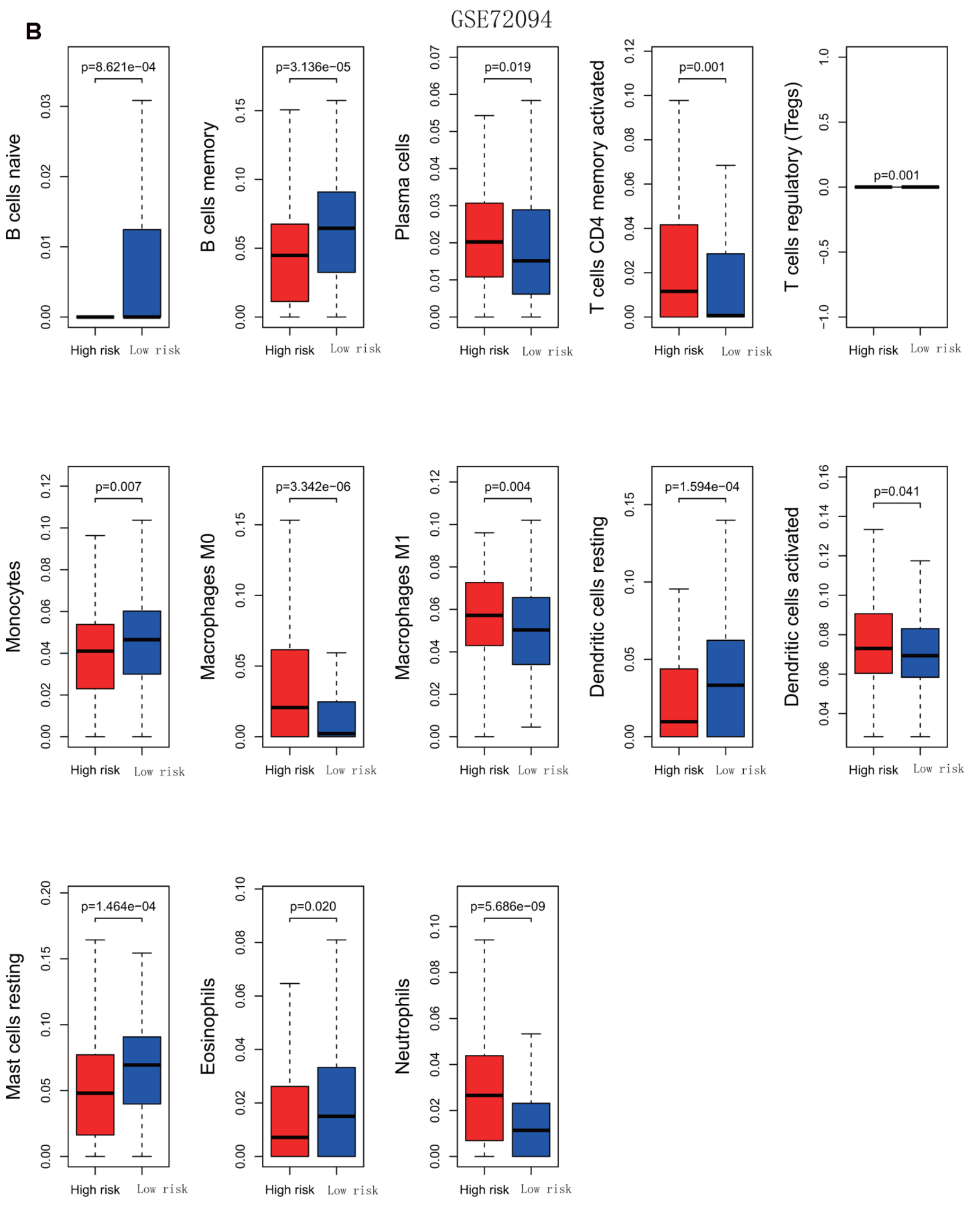

Supplementary Figure 6. Comparison of relative immune cell enrichment levels based on the CIBERSORT analysis results. (**A**, **B**) Bar plots show the statistically significant difference in the enrichment levels of immune cells between the high (red color) and low (blue color) risk groups in two LUAD cohorts: TCGA (**A**) and GSE72094 (**B**).

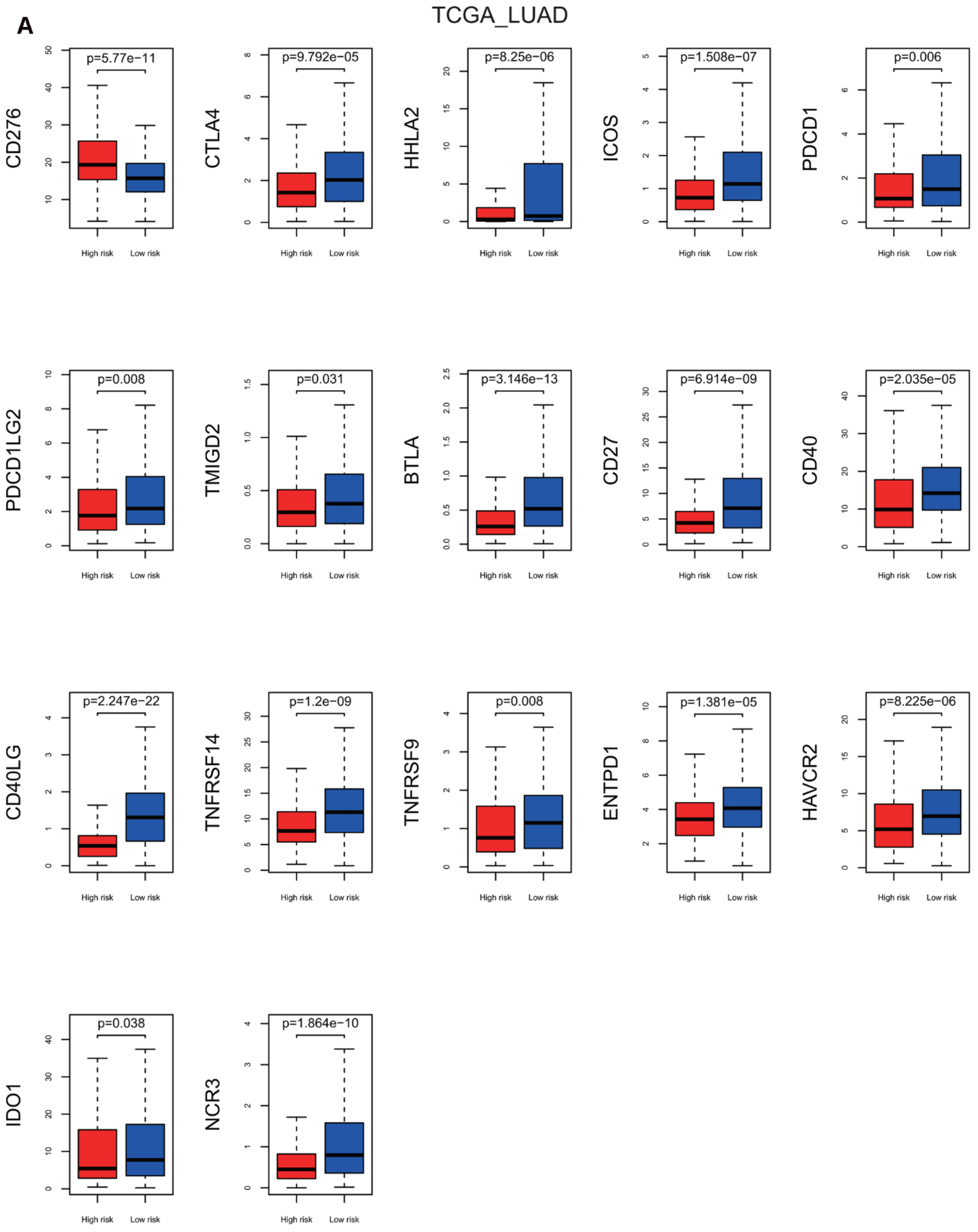
A
TCGA_LUAD
CD276
p=5.77e−11
CTLA4
p=9.792e−05
HHLA2
p=8.25e−06
ICOS
p=1.508e−07
PDCD1
p=0.006
PDCD1LG2
p=0.008
TMIGD2
p=0.031
BTLA
p=3.146e−13
CD27
p=6.914e−09
CD40
p=2.035e−05
CD40LG
p=2.247e−22
TNFRSF14
p=1.2e−09
TNFRSF9
p=0.008
ENTPD1
p=1.381e−05
HAVCR2
p=8.225e−06
IDO1
p=0.038
NCR3
p=1.864e−10
High risk
Low risk

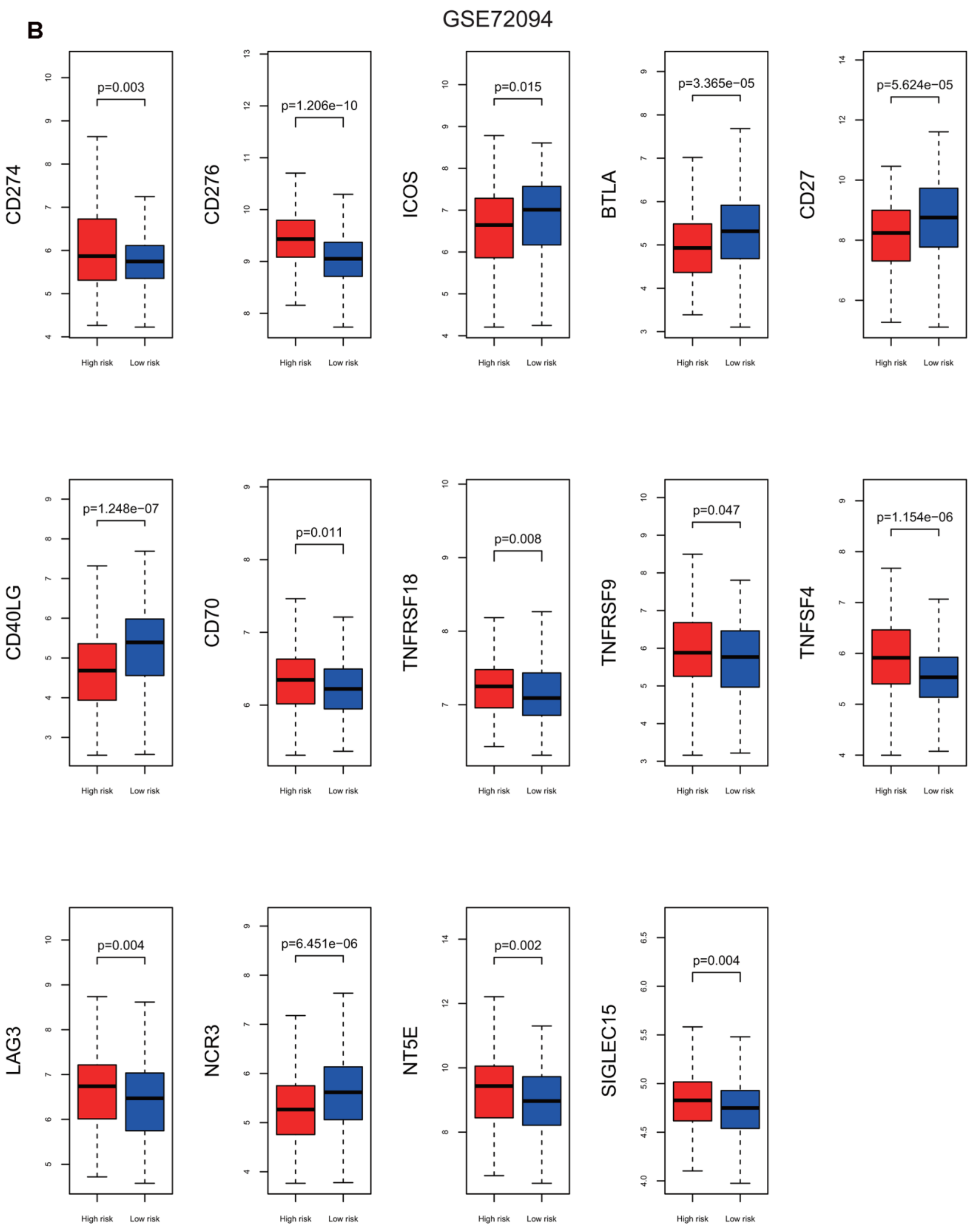

Supplementary Figure 7. Comparison of gene expression of 29 immune checkpoint genes in the LUAD cohorts. (**A**, **B**) Bar plots show the statistically significant difference in gene expression levels of immune checkpoint genes between the high (red color) and low (blue color) risk groups in two LUAD cohorts: TCGA (**A**) and GSE72094 (**B**).

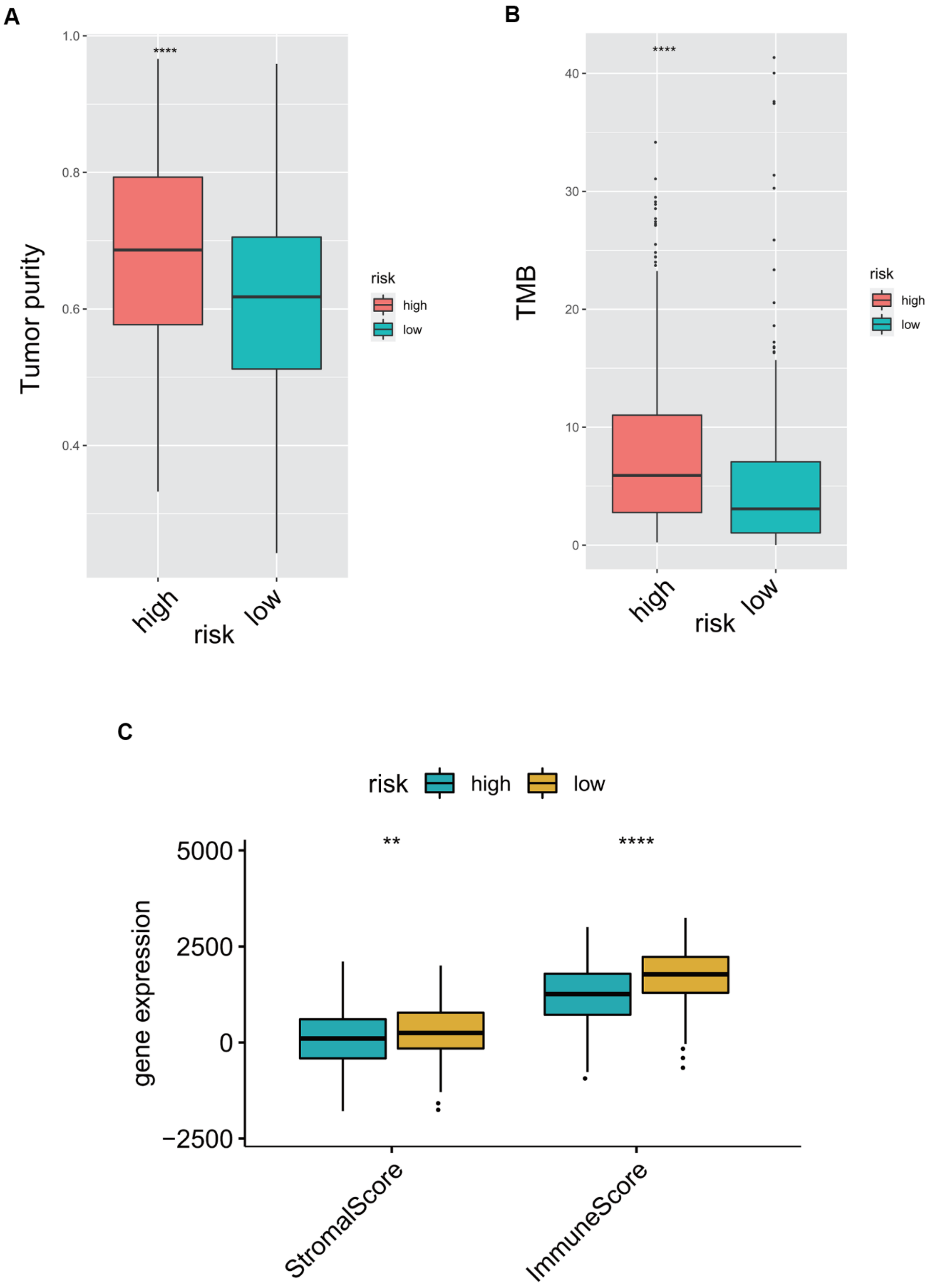

Supplementary Figure 8. Comparison of tumor purity, tumor burden, stromal score, or immune score between the high and low risk group in the TCGA LUAD cohort. (**A**, **B**) Bar plots show the comparison of tumor purity (**A**) and tumor burden (TMB) (**B**) between the high and low risk groups in the TCGA LUAD cohort. (**C**) Comparison of stromal score and immune score between the high and low risk group in the TCGA LUAD cohort.
