# Supplementary material for: Seven interferon gamma response genes serve as a prognostic risk signature that correlates with immune infiltration in lung adenocarcinoma
Source: Aging (Albany NY). 2021 Apr 4;13(8):11381–410. doi: 10.18632/aging.202831 (PMC8109098; doi:10.18632/aging.202831)
Supplement: Supplementary Tables [file aging-13-202831-s002.pdf]

SUPPLEMENTARY TABLES

Supplementary Table 1. Clinical features of two LUAD cohorts.

	Death (N=154)	alive (N=322)	Overall (N=476)
**Gender**			
Male	75 (48.7%)	144 (44.7%)	219 (46.0%)
Female	79 (51.3%)	178 (55.3%)	257 (54.0%)
**Age (years)**			
Mean (SD)	65.7 (10.7)	65.0 (9.69)	65.2 (10.0)
Median [Min, Max]	67.0 [40.0, 88.0]	66.0 [33.0, 87.0]	66.0 [33.0, 88.0]
**Stage**			
I	53 (34.4%)	206 (64.0%)	259 (54.4%)
II	46 (29.9%)	70 (21.7%)	116 (24.4%)
III	42 (27.3%)	37 (11.5%)	79 (16.6%)
IV	13 (8.4%)	9 (2.8%)	22 (4.6%)
**T**			
T1	39 (25.3%)	123 (38.2%)	162 (34.0%)
T2	87 (56.5%)	168 (52.2%)	255 (53.6%)
T3	18 (11.7%)	24 (7.5%)	42 (8.8%)
T4	10 (6.5%)	7 (2.2%)	17 (3.6%)

(a)Clinical features of the TCGA LUAD cohort.

	Death (N=109)	alive (N=280)	Total (N=389)
**Gender**			
Male	58 (53.2%)	114 (40.7%)	172 (44.2%)
Female	51 (46.8%)	166 (59.3%)	217 (55.8%)
**Age (years)**			
Mean (SD)	70.4 (8.54)	69.1 (9.75)	69.5 (9.43)
Median [Min, Max]	72.0 [46.0, 85.0]	70.0 [38.0, 89.0]	70.0 [38.0, 89.0]
**Stage**			
I	51 (46.8%)	201 (71.8%)	252 (64.8%)
II	24 (22.0%)	41 (14.6%)	65 (16.7%)
III	26 (23.9%)	31 (11.1%)	57 (14.7%)
IV	8 (7.3%)	7 (2.5%)	15 (3.9%)
**KRAS**			
Wildtype	64 (58.7%)	190 (67.9%)	254 (65.3%)
Mutation	45 (41.3%)	90 (32.1%)	135 (34.7%)
**EGFR**			
Wildtype	105 (96.3%)	244 (87.1%)	349 (89.7%)
Mutation	4 (3.7%)	36 (12.9%)	40 (10.3%)
**STK11**			
Wildtype	91 (83.5%)	234 (83.6%)	325 (83.5%)
Mutation	18 (16.5%)	46 (16.4%)	64 (16.5%)
**TP53**			
Wildtype	80 (73.4%)	215 (76.8%)	295 (75.8%)
Mutation	29 (26.6%)	65 (23.2%)	94 (24.2%)

(b)Clinical features of the GSE72094 LUAD cohort.

Supplementary Table 2. Analysis of 24 significantly differentially expressed interferon gamma response genes in the TCGA LUAD cohort.

Gene	conMean	treatMean	logFC	pValue	change
CD69	8.936670479	4.47653308	-0.997355581	3.19E-12	Down
CD74	1897.142334	1258.20791	-0.592457585	3.76E-10	Down
CD86	9.607033362	6.223707114	-0.626316828	4.52E-10	Down
CDKN1A	71.73071206	41.03490112	-0.805739475	6.18E-07	Down
CIITA	5.058851136	4.29529146	-0.236053757	0.00187674	Down
CSF2RB	9.875387254	5.517276061	-0.839881155	7.30E-15	Down
IL10RA	9.701275787	6.926449937	-0.486058378	2.96E-07	Down
IRF8	10.10959639	5.46877443	-0.88643594	2.31E-15	Down
LATS2	10.7276258	5.178535474	-1.050714763	1.56E-23	Down
LCP2	8.387419434	6.207701793	-0.434167749	4.55E-08	Down
MT2A	171.2990976	107.9445696	-0.666226884	0.000272554	Down
NOD1	4.764992505	4.034663216	-0.240025692	1.65E-05	Down
PTPN6	21.23585905	15.13571205	-0.488545923	6.81E-08	Down
SELP	10.56550028	2.27969876	-2.212445977	1.03E-29	Down
SOD2	31.28827632	26.96227591	-0.214679901	0.009578905	Down
IRF4	1.273380295	2.803021931	1.138319688	0.00081257	Up
ITGB7	1.223934115	1.669670931	0.448037896	0.03900192	Up
NMI	7.067112293	9.363642417	0.405949008	9.72E-05	Up
OAS3	8.38112837	16.96725326	1.017536638	1.93E-08	Up
PFKP	7.575956699	36.9750878	2.287053583	1.61E-24	Up
PNP	11.79940954	15.63021418	0.405622882	1.59E-05	Up
RBCK1	21.51999684	26.09297107	0.27798336	0.001820364	Up
RIPK2	7.642719185	10.60564426	0.472674335	0.000781467	Up
TRAFD1	13.22724719	16.44497774	0.314134211	3.75E-05	Up

Differential gene expression was analyzed using the 'limma' R package. Information, including gene name, mean gene expression in normal (conMean) or cancer (treatMean) samples, log fold change (logFC) of cancer vs. normal, p value (pValue) of logFC, and gene expression change pattern in cancer, were listed in the table.
